# Hypoxia-induced H19/YB-1 cascade modulates cardiac remodeling after infarction

**DOI:** 10.7150/thno.35218

**Published:** 2019-08-21

**Authors:** Oi Kuan Choong, Chen-Yun Chen, Jianhua Zhang, Jen-Hao Lin, Po-Ju Lin, Shu-Chian Ruan, Timothy J. Kamp, Patrick C.H. Hsieh

**Affiliations:** 1 Taiwan International Graduate Program in Molecular Medicine, National Yang-Ming University and Academia Sinica, Taipei, Taiwan; 2 Institute of Biomedical Sciences, Academia Sinica, Taipei, Taiwan; 3 Department of Medicine and Stem Cell and Regenerative Medicine Center, University of Wisconsin-Madison, WI, USA; 4 Institute of Medical Genomics and Proteomics and Department of Surgery, National Taiwan University and Hospital, Taipei, Taiwan

**Keywords:** Long noncoding RNA, extracellular matrix, cardiac remodeling, fibrosis

## Abstract

***Rationale:*** Long non-coding RNA (lncRNAs) has been identified as a pivotal novel regulators in cardiac development as well as cardiac pathogenesis. lncRNA H19 is known as a fetal gene but it is exclusively abundant in the heart and skeletal muscles in adulthood, and is evolutionarily conserved in humans and mice. It has been reported to possess a significant correlation with the risk of coronary artery diseases. However, the function of H19 is not well characterized in heart.

***Methods:*** Loss-of-function and gain-of-function mouse models with left anterior descending coronary artery-ligation surgery were utilized to evaluate the functionality of H19* in vivo*. For mechanistic studies, hypoxia condition were exerted in *in vitro* models to mimic cardiac ischemic injury. Chromatin isolation by RNA immunoprecipitation (ChIRP) was performed to reveal the interacting protein of lncRNA H19.

***Results:*** lncRNA H19 was significantly upregulated in the infarct area post-surgery day 4 in mouse model. Ectopic expression of H19 in the mouse heart resulted in severe cardiac dilation and fibrosis. Several extracellular matrix (ECM) genes were significantly upregulated. While genetic ablation of H19 by CRISPR-Cas9 ameliorated post-MI cardiac remodeling with reduced expression in ECM genes. Through chromatin isolation by RNA purification (ChIRP), we identified Y-box-binding protein (YB)-1, a suppressor of Collagen 1A1, as an interacting protein of H19. Furthermore, H19 acted to antagonize YB-1 through direct interaction under hypoxia, which resulted in de-repression of Collagen 1A1 expression and cardiac fibrosis.

***Conclusions:*** Together these results demonstrate that lncRNA H19 and its interacting protein YB-1 are crucial for ECM regulation during cardiac remodeling.

## Introduction

Myocardial ischemia not only triggers cardiomyocyte death but also launches a robust cardiac plasticity response that results in cardiac remodeling and fibrosis 1, 2. During this process, fibroblasts are activated, and excessive extracellular matrix (ECM) components, especially collagen type I and III, are over-produced and deposited in the destructed area to compensate for the loss of cardiomyocytes and to sustain the structural integrity of the myocardium 3, 4. However, this dysregulation of ECM homeostasis leads to deteriorated cardiac function with increased cardiac stiffness, and impaired contractility and electromechanical activity, which collectively ultimately lead to heart failure 5-7.

A hypoxic microenvironment is created during myocardial ischemia. The decrease in oxygen supply in the myocardium disrupts oxygen homeostasis. This stimulates the expression of hypoxia-inducible factor-1 (HIF-1), a key mediator of transcriptional responses to hypoxia. It activates more than 800 target genes involved in different signaling pathways such as cell apoptosis, proliferation, metabolism and angiogenesis 8. HIF-1 consists of two subunits, α-subunit, which is oxygen regulated, and β-subunit, which is expressed constitutively in the nucleus 9. HIF-1α is able to mediate cardioprotection induced by ischemic preconditioning; however, prolonged hypoxic conditions can lead to aberrant ventricular remodeling and cardiac fibrosis 10-12.

Over the past few decades, considerable research has been aimed at understanding the underlying mechanisms behind cardiac remodeling. However, most studies have focused on protein coding genes, which only occupy 2% of the human genome. The remnants (approximately 80% of noncoding RNAs) are less explored although they are actively transcribed 13-15. Long noncoding RNAs (lncRNAs) are known to have an important role in cardiac development, and lately, high-throughput RNA sequencing has been extensively utilized to profile and explore the transcriptome landscape of lncRNAs in failing hearts 16-19. Such studies have revealed that lncRNAs are mostly dysregulated in failing hearts and their expression signature can discriminate failing hearts of different etiologies 18. Moreover, recent studies have uncovered a critical role for lncRNAs in modulating cardiac remodeling and fibrosis 20-23. These studies demonstrate that lncRNAs play a crucial role in cardiac pathogenesis.

Recently, we found an upregulation of the lncRNA H19 at the infarcted area after myocardial infarction (MI) in a murine model, an experimental model for cardiac remodeling and fibrosis, suggesting lncRNA H19 plays a crucial role during cardiac pathogenesis. H19 is known as a fetal gene. It is highly expressed during embryonic development and is down-regulated after birth 24. A study of the lncRNA transcriptome performed on ischemic heart failure revealed that H19 is also abundantly expressed in mouse hearts 16. Human genome-wide association studies (GWAS) demonstrated that the H19 loci are correlated with high blood pressure in European ancestry 25. Furthermore, an H19 polymorphism was shown to possess a significant correlation with the risk of coronary artery diseases 26. These studies drive our attention towards deciphering the function of H19 in cardiovascular diseases.

Here, we investigated the role of H19 after infarction and explored its interacting proteins, particularly those responses to cardiac remodeling and fibrosis. Furthermore, loss-of-function and gain-of-function models were utilized to determine the functional role of H19 in heart failure progression.

## Materials and methods

### Animals

H19 knockout (H19^-/-^) mice were generated with assistance from the Transgenic Core Facility of the Institute of Molecular Biology, Academia Sinica using a CRISPR-Cas9-mediated genome editing approach. Two sgRNAs were designed to target H19 gene from 142,575,698 to 142,577,978 at the chromosome 7 of C57BL/6 mice. The sequences of the sgRNAs are 5′ GCAGAGCAAAGGCATCGCAA 3′ and 5′ TGTCGTCCATCTCCGTCTGA 3′. Heart-specific H19 overexpression or YB-1 knockdown C57BL/6 mice were intraperitoneally (IP) injected with 10^11^ viral genome (vg) AAV9 particles on postnatal day 8-12. All animals were housed under standard laboratory conditions in the animal core facility at Academia Sinica and all study protocols were approved by the Academia Sinica Institutional Animal Care and Utilization Committee in accordance with IACUC guidelines (IACUC No. 106083 and 11-09-211). All experimental mice used in this study were 8-10 weeks old male mice.

### Myocardial infarction model

Left anterior descending coronary artery-ligation surgery and sham surgery were performed according to a previous study 27. In brief, mice were anesthetized with 3% isoflurane followed by tracheal intubation using a 20G intravenous catheter and ventilated with a mixture of O_2_ and 1.5-2% isoflurane. Thoracic movement was checked to assure good ventilation. The respiration rate was adjusted to 120 min^-1^ with an inspiratory pressure of 17 to 18 cm H_2_O. Approximately 1.5 cm left side thoracotomy between the third and fourth ribs was performed and the thorax was opened carefully. LAD was permanently ligated with one single suture using sutures 6-0 polypropylene while for the control group, a suture was passed through the LAD but not ligated. Thorax was squeezed to remove air and the thoracic incision was closed layer by layer using running sutures. The endotracheal tube was removed and running sutures were performed on cervical incision as well. A successful LAD ligation-induced MI was characterized by transthoracic two-dimensional echocardiography one day after surgery. The same procedure was performed as sham surgery without the ligation.

### Echocardiography

Echocardiography was performed and analyzed using Vivid-q Ultrasound (GE) equipped with a 5.0-13.0 MHz intraoperative probe. A mouse was anesthetized with 2% isoflurane mixed with 0.5 L/min 100% O_2_. Then, the mouse was fixed in a supine position with ECG leads embedded, to monitor heartbeat rate and rhythm. The anesthesia system was adjusted to 0.5-1% isoflurane mixed with 0.5 L/min 100% O_2_ in order to maintain a steady-state sedation level a with heart rate of 450 ± 50 beats per min (bpm). A layer of ultrasound gel was applied on the chest of the mouse once it was under steady-state sedation level. Then, two-dimensional (2D) imaging (B-mode) was performed to obtain the parasternal short axis view using Vivid-q Ultrasound (General Electric Company) equipped with a 5.0-13.0 MHz intraoperative probe. In B-mode orientation, the left ventricle, including papillary muscles and septal wall; and a slight portion of right ventricular wall were observed. Next, a one-dimensional (1D) imaging (M-mode) was recorded to measure the cardiac dimensions and contractility. Echo images were further analyzed using echo work station to measure and analyze ejection fraction (EF) and fraction shortening (FS).

### Cardiac catheterization

Cardiac catheterization was performed according to a previous study to measure the ventricular pressure-volume relationship in mice 28. A mouse was anesthetized with urethane (800 mg/kg) and fixed in a supine position on the heating pad with the temperature set to 37 °C. Tracheal intubation was performed on the mouse and supplied with 100% O_2_. The PV catheter was inserted to the LV through right carotid artery without opening the chest cavity. The systolic and diastolic heart functions were measured and analyzed using Millar SPR-839 instrument and LabChart Pro analysis software, respectively. The systolic and diastolic functional measurements such as the peak rate of pressure rise (dP/dt_max_), preload recruited stroke work (PRSW), end-systolic pressure-volume relation (ESPVR), peak rate of pressure decline (dP/dt_min_), relaxation time constant (Tau) and end-diastolic PV relation slope (EDPVR) were determined by the analysis software.

### Isolation of cardiomyocyte and cardiac fibroblast

Neonatal mouse cardiomyocytes and cardiac fibroblasts were isolated as described 29. Neonatal mice hearts were finely minced into 1 mm pieces. The minced hearts were digested with digestion buffer (1 mg/ml collagenase type II [Worthington Biochemical Corporation] in Hank's balanced salt solution) at 37 °C with gentle rocking. The dissociated cells were pre-plated for 1 h at 37 °C to separate cardiac fibroblasts from cardiomyocytes. Adult mouse cardiomyocytes and cardiac fibroblasts were isolated as described 30, 31. To isolate adult cardiac fibroblasts, adult mouse hearts were finely minced using a sterile razor blade to 2 mm pieces. The minced hearts were digested with digestion buffer (2 mg/ml collagenase type IV [Worthington Biochemical Corporation] and 1.2 U/ml dispase II [Sigma-Aldrich] in Dulbecco's phosphate-buffered saline) at 37 °C for 15 min with gentle rocking. The digestion buffer containing the tissue was triturated by pipetting. The suspensions were filtered using a 40 μm cell strainer. The filtered cell suspensions were centrifuged at 200 g for 20 min to remove tissue debris. Cell pellets were resuspended in DPBS containing 2% FBS before antibody staining. The cardiac fibroblasts were sorted out from non-myocytes using anti-DDR2 antibody (GeneTex, GTX102526) or anti-PDGFR-α antibody (ThermoFisher Scientific, 12-1401-81). A Langendorff-free method was used to isolate adult cardiomyocytes. The mouse was anaesthetized and the chest cavity was opened to fully expose the heart. The descending aorta and inferior vena cava were cut followed by injection of EDTA buffer into the apex of the right ventricle. Sequential injection of EDTA buffer, perfusion buffer and collagenase buffer were performed to digest the clamped heart. The heart was gently pulled into pieces and dissociated by gentle trituration.

### Cell line and cell culture

Mouse embryonic fibroblast cell line (NIH3T3), purchased from the Bioresource Collection and Research Center, Taiwan, was cultured in Dulbecco's Modified Eagle Medium, high glucose (Gibco) supplemented with 10% bovine serum (Gibco) and 1x penicillin/streptomycin (Corning). Differentiation and maintenance of human iPSC-Cardiac fibroblasts (hiPSC-CFs) were carried out as described in Zhang et al 32. In brief, human iPSCs were dissociated and seeded on Matrigel (GFR, BD Biosciences) coated 6-well plates in mTeSR1 medium supplemented ROCK inhibitor (Y-27632) (Tocris). Cells were cultured for 5-6 days in mTeSR1 medium with medium changes daily until they reached 100% confluence when differentiation started. The medium was then changed to RPMI+B27 (Gibco) without insulin and supplemented with CHIR99021 (Tocris) for 24 h, followed with CFBM medium supplemented with bFGF until day 20 when they were used for flow cytometry analysis and passaged. The hiPSC-CFs were fed every other day with the FibroGRO+2% FBS medium and passaged every 4-6 days using 0.05% Trypsin-EDTA. For the hypoxia experiments, cells were incubated in a hypoxia chamber supplemented with 1% O_2_ and 5% CO_2_ for 48 h.

### Chromatin isolation by RNA purification

Chromatin isolation by RNA purification (ChIRP) was performed according to a previous protocol with modification 33. Antisense DNA tiling probes targeting lncRNA H19 were designed using the online probe designer at singlemoleculefish.com (Table S1). LacZ was selected as control RNA in this experiment. Cells were fixed with 1% glutaraldehyde (Sigma-Aldrich) and the cross-linking reaction was quenched with glycine. The cell pellets were sonicated in Bioruptor (Diagenode) to obtain DNA size 200-800 bp. The lysate was hybridized with probes and pulled down using magnetic beads (Invitrogen). Beads were washed with wash buffer and re-suspended in DNase buffer (100 mM NaCl and 0.1% NP-40). Protein eluent was further processed for mass spectrometry analysis. Mass spectrometry analysis was performed with the assistance from the Proteomics Core in the Institute of Biomedical Sciences, Academia Sinica. The proteins identified in this experiment are listed in Table S2.

### Chromatin immunoprecipitation (ChIP)

The experimental procedures were performed according to the manufacturer's protocol (Active Motif). A total of 1 x 10^7^ cells were cross-linked with 1% paraformaldehyde. The fixation reaction was quenched and the cells were lysed with lysis buffer. The genomic DNA was sheared into smaller fragments (200-800 bp) by enzymatic digestion. A rabbit anti-YB-1 antibody (Abcam, ab12148) was added into the sample to immunoprecipitate the protein-DNA complex of interest. The crosslinking of protein-DNA complex was reversed and DNA was purified from the sample using a chromatin IP DNA purification kit (Active Motif) and subjected to real-time quantitative PCR analysis. The primers used for ChIP-qPCR are listed in Table S3.

### Real-time quantitative PCR (qPCR)

Total RNA from different samples was extracted by Trizol reagent (Invitrogen). Total RNA was reverse transcribed into cDNA using SuperScript III Reverse Transcriptase (Invitrogen). Real-time quantitative PCR was performed using OmicsGreen qPCR Master Mix (Omics Bio) with cDNA serving as template. The reactions were carried out in an ABI 7500 real-time PCR machine (Applied Biosystems). The primers used are listed in Table S3.

### Total collagen assay

The experimental procedures were conducted according to the manufacturer's protocol (BioVision). Mouse hearts were collected and homogenized in water. The samples were hydrolyzed with concentrated HCl at 120 °C for 3 h. Chloromine T reagent was added into the samples followed by DMAB reagent and incubated for 90 min at 60 °C. The samples were measured at 560 nm absorbance.

### Immunoblotting

Samples were lysed with RIPA lysis buffer (50 mM HEPES, pH7.5; 140 mM NaCl; 1 mM EDTA; 1% Triton X-100 and 0.1% SDS) with protease inhibitor (Sigma). Total proteins were collected and quantified using Bio-Rad protein assay (Bio-Rad). Total proteins were heat denatured and separated in the SDS-PAGE gel electrophoresis system. Proteins of interest were studied by hybridizing with corresponding antibodies such as rabbit anti-COL1A1 (GeneTex, GTX112731), rabbit anti-YB-1 (Millipore, ABE187), rabbit anti-HIF-1α (Proteintech, 20960-1-AP), mouse anti-Gapdh (Millipore, MAB374).

### RNA immunoprecipitation (RIP)

Cells were lysed with hypotonic lysis buffer (10 mM Tris pH7.5, 10 mM NaCl, 10 mM EDTA, 0.5% TritonX-100, proteinase inhibitor). The lysate was centrifuged and the supernatant was pre-cleaned with rabbit anti-IgG (Millipore). After pre-cleaning, rabbit anti-IgG or rabbit-anti-YB-1 (Abcam, ab12148), together with protein G magnetic beads were added into the samples and incubated at 4 °C for 16-18 h. The magnetic beads were removed and washed with wash buffer (50 mM Tris pH7.5, 150 mM NaCl, 0.05% NP-40). The beads were re-suspended in proteinase K buffer (100 mM NaCl, 10 mM Tris-Cl pH7.0, 1 mM EDTA, 0.5% SDS, proteinase K). Then, RNAs were extracted using the Trizol method.

### RNA electrophoretic mobility-shift assay (RNA-EMSA)

RNA-EMSA was performed according to the manufacturer's protocol (Thermo Scientific). A short fragment of H19 RNA (85 bp) was *in vitro* transcribed (Promega) and 3′ end biotinylated (Thermo Scientific). YB-1 protein was *in vitro* translated using 1-step human coupled IVT kit (Thermo Scientific). The binding reactions were carried out by adding both H19 RNA and YB-1 protein in binding reaction buffer (Thermo Scientific). The samples were electrophoresed in 6% polyacrylamide gel and transferred to nylon membrane (PerkinElmer). The RNAs were visualized using UVP BioSpectrum Imaging Systems. The dissociation constant (Kd) was evaluated by performing a binding reaction in serial-diluted protein.

### Lentivirus-based shRNAs expression and siRNAs

All the shRNAs were purchased from the National RNAi Core Facility, Academia Sinica. These shRNAs were constructed in pLKO.1 lenti-based expression vector and the lentiviruses for the shRNAs were produced according to the protocol provided. Cells were infected with lentivirus and selected by puromycin (Sigma) for several days. The knockdown efficiency was assessed by qPCR and immunoblotting. The target sequence of shRNAs that applied in experiments were shYbx1-1: GAGAACCCTAAACCACAAGAT, shYbx1-2: GTATCGCCGAAACTTCAATTA, shYbx1-3: GTATCGCCGAAACTTCAATTA and control shLuciferase: GCGGTTGCCAAGAGGTTCCAT. All the siRNAs were purchased from ThermoFisher Scientific, and transfected accordingly into the cells using siRNA transfection reagent (SignaGene Laboratories, SL100566).

### Immunofluorescence of total secreted extracellular matrix (ECM)

NIH3T3 cells were seeded into 8 well chamber slides and cultured for 8 days with the medium changed every two days. Cells were removed by incubating the cells in 20 mM ammonium hydroxide. The insoluble ECM was fixed with 2% paraformaldehyde and hybridized with a rabbit anti-COL1A1 (GeneTex, GTX112731).

### Luciferase assay

The upstream of COL1A1 promoter including 5′ UTR (-884 ~ +148), COL1A2 promoter (-530 ~ +243), COL3A1 promoter (-1238 ~ +242) and Fn1 promoter (-1245 ~ +251) were cloned into pGL3-basic vector (Promega) as reporter plasmids. Plasmids (pGL3-basic, pGL3-Col1a1, and pRluc-C1) and siRNAs (siCtrl and siYB-1) (ThermoFisher Scientific) were co-transfected into NIH3T3 cells to achieve different conditions (YB-1 knockdown, H19 knockout or H19-YB-1 double knockdown) using X-tremeGENE HP DNA Transfection Reagent (Roche). Luciferase activities were assayed by the Dual-Luciferase Reporter Assay System (Promega) according to the manufacturer's protocol.

### Recombinant Adeno-Associated Virus (AAV)

For AAV-H19OE, full length of lncRNA H19 was constructed and cloned into the pAAV-U6-GFP plasmid (Vigene Bioscience). Then, shRNAs for YB-1 deficiency experiments were sub-cloned from pLKO.1 lenti-based expressing vector into pAAV-U6-GFP plasmid as well. HEK293T cells were co-transfected with three plasmids pHelper, pXX9 and expression plasmid (pAAV-U6-GFP, pAAV-U6-H19OE, pAAV-U6-shluciferase and pAAV-U6-shYB-1) using the calcium phosphate transfection method. AAV particles were purified by cesium chloride gradient centrifugation and concentrated using Vivaspin 20 centrifugal concentrators 100K MWCO (Vivascience Inc.). The virus titer was determined by real-time PCR.

### Immunohistochemistry and immunofluorescent staining

Heart tissues were obtained at 4 days after MI or sham surgery. The samples were fixed in 4% paraformaldehyde and embedded in paraffin for sectioning. Sections underwent deparaffinization and rehydration for Masson's trichorme staining. Masson's trichrome staining was utilized to evaluate collagen deposition. The Masson's trichrome staining was applied on the deparaffinized and rehydrated sections according to manufacturer's protocol (Sigma). The percentage of the LV fibrosis area was measured using ImageJ and the LV fibrosis area was expressed as the percentage of LV fibrosis area over total LV area 34. For immunofluorescent staining, heart sections were deparaffinized and rehydrated as mentioned above. Serum blocking were performed for 30 min and incubated with primary antibody such as mouse anti-Vim (Sigma-Aldrich, V2258), rabbit anti-YB-1 (Millipore, ABE187), rabbit-anti H3P (Millipore, 04-1093), mouse anti-GFP (MBL, M048-3), rabbit anti-DDR2 (GeneTex GTX102526) for overnight at 4 °C. After washing, sections were incubated with Alexa Fluor 488 or Alexa Fluor 594-conjugated secondary antibody (Invitrogen) and DAPI. Images were taken by confocal microscope (Model-Zeiss LSM 700).

### Triphenyltetrazolium chloride (TTC) staining

Mice with MI surgery at day 4 in different experimental groups were sacrificed and the heart was sliced into five sections of 1.0 mm thickness each. The sections were incubated with 1% triphenyltetrazolum chloride in phosphate solution (TTC, Sigma) for 15 min at room temperature. After 15 mins, the sections were arranged and then digitally photographed. The infarct size was analyzed using ImageJ and the infarct size was expressed as the percentage of infarct length over total LV circumference 35.

### Cell proliferation assay

Cell proliferation assay was carried out according to the manufacturer's protocol (Invitrogen). Click-iT EdU Alexa Fluor 647 Flow Cytometry Assay Kit was utilized to measure DNA synthesis directly using a flow cytometer. Cells were incubated with Edu by adding Edu to the culture medium for 2 h. Then, cells were harvested and fixed. The DNA content was measured by flow cytometer (Becton Dickinson, LSRII SORP).

### Cell apoptosis assay

Cell apoptosis was evaluated using Annexin V kit (Invitrogen) and processed according to the manufacturer's protocol. Cells were harvested and incubated with annexin V conjugates. Then, the cells were analyzed by flow cytometer (Becton Dickinson, LSRII SORP).

### Statistical analysis

GraphPad Prism 5 (GraphPad Software, La Jolla, USA) was utilized to perform statistical analysis of every experiment. Data are presented as mean ± SEM. Comparison between two groups was analyzed using Student's* t*-test while differences among multiple groups was analyzed by one-way ANOVA. P values of less than 0.05 were considered significant.

## Results

### Overexpression of lncRNA H19 exacerbates cardiac dilation and fibrosis after injury

**Figure 1:**
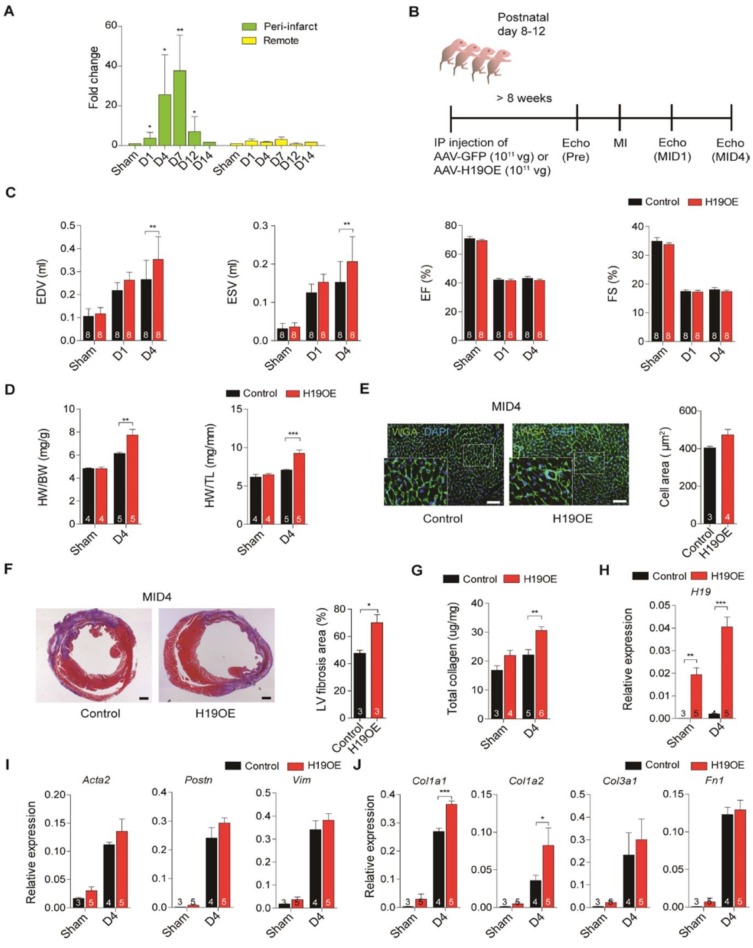
** Ectopic overexpression of H19 exacerbates cardiac dilation and fibrosis after injury. (A)** Dynamic expression of H19 in the peri-infarct and remote areas post-MI (n=3). Data represent means ± SEM, *P < 0.05, **P < 0.01, one-way ANOVA. **(B)** Diagram of experimental design. At postnatal day 8 to 12, mice were I.P. injected with a single dose of 1 x 10^11^ vg of AAV9-GFP (control) or AAV-H19OE (H19OE). LAD-ligation surgery and sham surgery (without ligation) were performed on 8-week-old mice in both the control and H19OE groups. A series of echocardiographies were performed post-MI to evaluate cardiac functions. **(C)** Echocardiography analysis, EDV (end diastolic volume), ESV (end systolic volume), EF (ejection fraction) and FS (fraction shortening), on control and H19OE mice at post-MI day 1 (D1) and day 4 (D4). Data represent means ± SEM, **P < 0.01, one-way ANOVA. **(D)** Evaluation of heart weight to body weight and heart weight to tibia length ratios in mice at post-MI D4. Data represent means ± SEM, **P < 0.01, ***P < 0.001, one-way ANOVA. **(E)** Representative photomicrographs of immunofluorescent staining of wheat germ agglutinin (WGA), and DAPI in myocardial tissue sections, scale bar: 50 μm. Data are expressed as mean ± SEM, Student's t-test. **(F)** Representative images of Masson's trichrome staining of the whole heart after MI, scale bar: 500 µm. Data are shown as mean ± SEM, *P < 0.05, Student's t-test. **(G)** Evaluation of total collagen in the heart at post-MID4. Data represent means ± SEM, **P < 0.01, one-way ANOVA. **(H-J)** Relative expression of (H) H19, (I) fibrosis markers Acta2, Postn and Vim and and (J) extracellular matrix components Col1a1, Col1a2, Col3a1 and Fn1 post-MID4. Data are expressed as mean ± SEM, *P < 0.05, **P < 0.01, ***P < 0.001, one-way ANOVA.

The re-expression of lncRNA H19 in ischemic heart failure has not merely been observed in a mouse model but also in patients 16, 36. This suggests that lncRNA H19 may play an important role in cardiovascular diseases. To assess the biological role of lncRNA H19 in the heart, we first established the lncRNA H19 expression profile in the heart. We found that lncRNA H19 was exceptionally enriched in the heart and skeletal muscles (Figure S1). To investigate the function of lncRNA H19 in the heart during disease development, we examined the expression of lncRNA H19 in mouse hearts upon ligation of the left anterior descending coronary artery. Intriguingly, we observed a re-expression of lncRNA H19 that was only detected in the peri-infarct area but not in the remote area, especially in the early period post-MI, day 4 (D4) to day 7 (D7) (Figure 1A). According to a literature review, cardiac remodeling starts at approximately day 3 post-MI right after acute inflammation and is sustained until a month post-MI in a mouse model 37. Additionally, fibroblasts are activated and highly proliferative within 2 to 4 days post-MI and the extracellular matrix proteins are abundantly expressed starting from day 3 to day 7 post-MI 38. Since the time window of H19 upregulation falls within day 1 to day 7 post-MI, we hypothesized that H19 might exert its function during the early stage of cardiac remodeling. To study the function of lncRNA H19, a single dose of 1 × 10^11^ viral genome of Adeno-Associated Virus 9 (AAV9) - GFP or AAV-H19 overexpression (OE) were intraperitoneally injected into postnatal 8-12-day-old mice to overexpress lncRNA H19 specifically in the heart 39. The impact of lncRNA H19 overexpression on the mice was examined through MI surgery. A series of echocardiography analysis was conducted on 8-week-old mice before MI, one day (D1) and four days (D4) post-MI to monitor cardiac functions in mice (Figure 1B). We observed a robust expression of lncRNA H19 in the heart of 8-week-old mice compared to the control (Figure S2A). Cardiac chamber dilation and heart functions were evaluated in both the control and H19OE groups, and we found out that without MI, no significant differences were observed in end-diastolic volume (EDV), end-systolic volume (ESV), ejection fraction (EF) and fraction shortening (FS) (Figure S2B). The heart weight was also indistinguishable (Figure S2C). Intriguingly, we noticed that the EDV and ESV were significantly larger in H19OE mice post-MID4 compared to control mice (Figure 1C and Table S4), indicating that H19OE mice underwent severe cardiac dilation at the early stage of cardiac remodeling. The isolated hearts from H19OE mice at post-MI D4 showed a significant increase in heart weight (Figure 1D). Furthermore, we observed a slight increase in the size of cardiomyocytes in H19OE mice post-MID4, suggesting the overexpression of H19 indeed has a mild effect on cardiac hypertrophy (Figure 1E). We also noticed a significant increase of the infarct area in the H19OE hearts compared to control hearts after injury (Figure S3). Interestingly, Masson's trichrome staining of heart sections revealed that severe fibrosis manifested in the H19OE hearts after injury (Figure 1F). Moreover, the total collagen content was substantially up-regulated in H19OE hearts after injury (Figure 1G), indicating severe collagen deposition in H19OE hearts. To verify whether overexpression of lncRNA H19 persisted during MI, we examined the RNA level of H19 in sham and post-MI D4. We observed a similar expression pattern of lncRNA H19 between both groups but with greater magnitude in the H19OE group compared to the control group (Figure 1H). In addition, we also observed a significant increase in fibrosis markers 40, 41 such as α-smooth muscle actin (Acta2), periostin (Postn) and vimentin (Vim) in both control and H19OE mice at D4 post-MI compared to sham and an upregulation trend in H19OE mice compared to the control at the same time point (Figure 1I). Intriguingly, H19OE mice revealed a significant upregulation in collagen type I alpha 1 (Col1a1) and collagen type I alpha 2 (Col1a2) and an increasing trend in collagen type III alpha 1 (Col3a1) and fibronectin (Fn1), suggesting that H19OE mice underwent severe ECM remodeling (Figure 1J). Of note, several studies utilizing AAV as gene delivery system have pointed out that AAV preferentially transduces to cardiomyocytes 42, 43. However, we observed GFP and DDR2 double-positive cells in the infarcted area, which suggests that the GFP proteins were expressed in cardiac fibroblasts (Figure S4A). Through cell sorting of PDGFR-α^+^ cells, we confirmed that AAV9 is also capable of transducing to cardiac fibroblasts with approximately 7 fold upregulation of H19 compare to the control (Figure S4B). Together these results suggest that H19 facilitates cardiac dilation, fibrosis and ECM-related gene expression at the early stage post-MI.

### Ablation of lncRNA H19 attenuates cardiac dilation and fibrosis after injury

**Figure 2:**
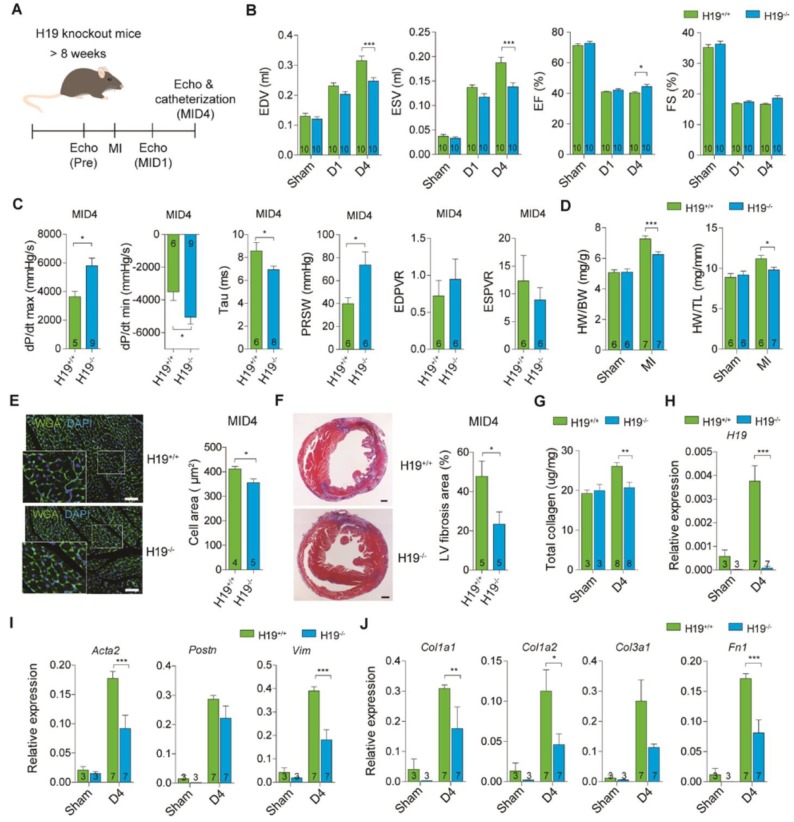
** Ablation of H19 attenuates cardiac dilation and fibrosis after injury. (A)** Diagram for experimental design. Eight-week-old mice of both wild type (H19^+/+^) and H19 knockout (H19^-/-^) groups underwent LAD-ligation surgery and sham surgery (without ligation), respectively. Echocardiography was performed post-MI D1 and D4. **(B)** Echocardiography analysis, EDV (end diastolic volume), ESV (end systolic volume), EF (ejection fraction) and FS (fraction shortening) after MI. Data represent means ± SEM, *P < 0.05, **P < 0.01, ***P < 0.001, one-way ANOVA. **(C)** Cardiac catheterization on mice post-MI day 4. Peak rate of pressure rise (dP/dt_max_), preload recruited stroke work (PRSW), end-systolic pressure-volume relation (ESPVR), peak rate of pressure decline (dP/dt_min_), relaxation time constant (Tau) and end-diastolic PV relation slope (EDPVR) were evaluated. Data represent means ± SEM, *P < 0.05, Student's t-test. **(D)** Heart weight to body weight and heart weight to tibia length ratios in mice after MI. Data represent means ± SEM, *P < 0.05, ***P < 0.001, one-way ANOVA. **(E)** Representative photomicrographs of immunofluorescent staining of wheat germ agglutinin (WGA), and DAPI in myocardial tissue sections, scale bar: 50 μm. Data are expressed as mean ± SEM, *P < 0.05, Student's t-test. **(F)** Representative images of Trichrome staining of whole heart post-MI D4, scale bar: 500 µm. Data are shown as mean ± SEM, *P < 0.05, Student's t-test. **(G)** Evaluation of total collagen in the heart post-MI D4. Data represent means ± SEM, ***P < 0.001, one-way ANOVA. **(H-J)** Relative expression of (H) H19, (I) fibrosis markers, Acta2, Postn and Vim and (J) extracellular matrix components Col1a1, Col1a2, Col3a1 and Fn1 post-MI D4. Data are expressed as mean ± SEM, *P < 0.05, **P < 0.01, ***P < 0.001, one-way ANOVA.

Earlier observations suggested that H19 overexpression exacerbates fibrosis progression, thus implicating H19 as a regulatory factor to induce ECM production and cardiac remodeling. To further validate this hypothesis, we then generated H19 knockout (KO) mice using a CRISPR-Cas9-mediated genome editing approach to study the functionality of H19 during early stage post-infarction (Figure S5). MI was performed on 8-week-old H19 KO mice, followed by a series of echocardiography analyses (Figure 2A). The H19 gene knockout was confirmed by quantitative PCR (qPCR) in the heart of homozygous H19^-/-^ mice (Figure S6A). H19^-/-^ mice did not show any differences in cardiac systolic and diastolic functions or heart weight in comparison with control littermates (Figure S6B-D). Intriguingly, we observed that the cardiac performance, and the systolic and diastolic functions in H19^-/-^ mice were ameliorated compared to H19^+/+^ mice post-MID4 (Figure 2B-C and Table S5). Furthermore, the hearts were less dilated in H19^-/-^ mice compared to H19^+/+^ mice post-MID4 (Figure 2D). These data demonstrate reduced adverse cardiac remodeling and improved heart function in H19^-/-^ mice post-MI. Pathological analysis of the heart morphology revealed that the cardiomyocytes in H19^-/-^ mice were less hypertrophic at post-MID4 (Figure 2E). After further histological analysis of the heart, we found that H19^-/-^ mice possessed smaller infarct size, less fibrosis area and less collagen deposition after MI, as revealed by TTC staining, Masson's trichrome staining and collagen assay (Figure 2F-G and Figure S7). To ensure that H19 was expressed accordingly, we tested the H19 expression in both H19^+/+^ and H19^-/-^ mice at post-MID4. The H19 expression was upregulated after injury in H19^+/+^ mice but not in H19^-/-^ mice (Figure 2H). Consistently, the expression of fibrosis marker and ECM genes was significantly downregulated in H19^-/-^ mice post-MI (Figure 2I-J). The above results were compatible with the findings in H19 overexpression mice, suggesting that H19 is indeed a key factor in regulating cardiac remodeling and fibrosis.

### H19 directly interacts with YB-1 in cardiac fibroblasts under hypoxia

**Figure 3:**
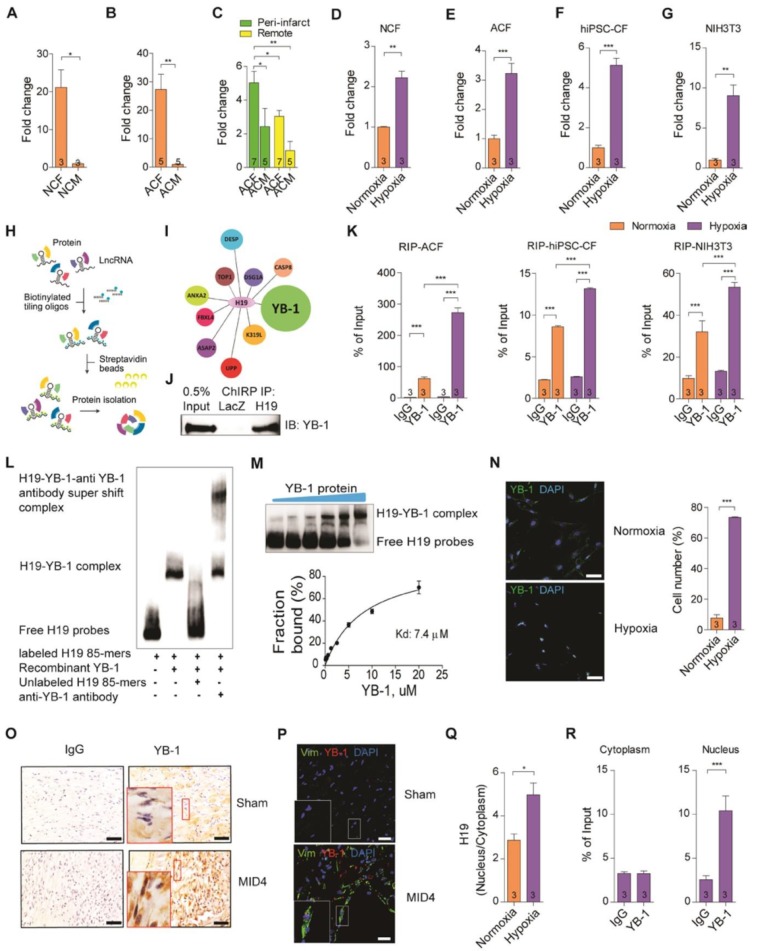
** H19 directly interacts with YB-1 in cardiac fibroblasts under hypoxia.** (A-B) Quantification of H19 in (A) neonatal cardiac fibroblasts (NCF) and neonatal cardiomyocytes (NCM), (B) adult cardiac fibroblasts (ACF) and adult cardiomyocytes (ACM). Data represent means ± SEM, *P < 0.05, **P < 0.01, Student's t-test. **(C)** Relative expression of H19 in different cell types including adult cardiac fibroblasts (ACF) and adult cardiomyocytes (ACM) in the peri-infarct and remote area post-MI day 7. Data represent means ± SEM, *P < 0.05, **P < 0.01, one-way ANOVA. **(D-G)** Relative expression of H19 in (D) neonatal cardiac fibroblasts (NCF), (E) adult cardiac fibroblasts (ACF), (F) human iPSC-derived cardiac fibroblasts (hiPSC-CF) and (G) NIH3T3 cells under normoxia and hypoxia conditions. Data represent means ± SEM, **P < 0.01, ***P < 0.001, Student's t-test. **(H)** Diagram showing chromatin isolation by RNA purification (ChIRP). LncRNA H19 was pulled down using biotinylated probes. **(I)** Mass spectrometry results showing proteins that interacted with H19. **(J)** Representative image of immunoblotting of YB-1 in ChIRP of H19, LacZ as control. **(K)** Detection of H19 after RNA immunoprecipitation of YB-1 by quantitative PCR in adult cardiac fibroblasts (ACF), human iPSC-derived cardiac fibroblasts (hiPSC-CF) and NIH3T3 cells under normoxia and hypoxia conditions. Data represent means ± SEM, ***P < 0.001, one-way ANOVA. **(L)** Representative image of electrophoretic mobility shift assay (EMSA) between H19 and YB-1. **(M)** Evaluation of YB-1 dissociation constant (Kd). **(N)** Representative images of immunostaining of YB-1 localization of NIH3T3 cells under hypoxia, YB-1 (green), nucleus (blue), scale bar: 50 µm. **(O)** Immunohistochemistry staining of YB-1 in mouse hearts at control (sham) and day 4 post-MI (MI4D), scale bar: 20 μm. **(P)** Representative immunofluorescent staining of YB-1 (red), Vim (green) and DAPI (blue), scale bar: 20 μm. Note YB-1 localization in the nucleus of cardiac fibroblasts from mouse hearts isolated on day 4 post-MI. **(Q)** Ratio of H19 distribution in the nucleus to cytoplasm under normoxic and hypoxic conditions measured by qPCR after subcellular fractionation. Data represent means ± SEM, *P < 0.05, one-way ANOVA. **(R)** Individual evaluation of H19-YB-1 interaction in the nucleus and cytoplasm after subcellular fractionation using RNA immunoprecipitation under hypoxia. H19 was detected using qPCR. Data represent means ± SEM, ***P < 0.001, one-way ANOVA.

The progression of heart failure is associated with the dysregulation of ECM properties, leading to an alteration in myocardial architecture and mechanics that severely impacts overall cardiac function 44. Emerging evidence suggests that the disruption of ECM in the heart is detrimental to and may also result in the deterioration of cardiac systolic and diastolic functions 45-47. We therefore hypothesized that these cardiac dysfunctions may underlie the effect of ECM dysregulation that is regulated by lncRNA H19 at the early stage post-MI. Cardiac fibroblasts are the major cell regulator of wound healing and ECM synthesis, generating a scaffold to support cardiac structure and function 48. We evaluated the expression of lncRNA H19 in different cell types, especially cardiomyocytes and cardiac fibroblasts isolated from neonatal and adult mice (Figure S8). Strikingly, we found that lncRNA H19 was extremely enriched in neonatal and adult cardiac fibroblasts (Figure 3A-B). In particular, we observed a significant upregulation of lncRNA H19 in adult cardiac fibroblasts and an increase in the trend for lncRNA H19 in cardiomyocytes isolated from the peri-infarct area but not from the remote area (Figure 3C). Collectively, these data suggest that lncRNA H19 is predominantly expressed in cardiac fibroblasts, highlighting H19 as a pivotal element in the regulation of ECM remodeling of the heart after injury. To mimic myocardial ischemia *in vitro*, we incubated the isolated neonatal cardiac fibroblasts (NCF) and adult cardiac fibroblasts (ACF) under hypoxic conditions. Strikingly, we observed a significant increase in lncRNA H19 expression in hypoxia-treated cells (Figure 3D-E). Furthermore, we were interested to examine the phenomena on human cardiac fibroblasts. Human induced pluripotent stem cell (iPSC)-derived cardiac fibroblasts (hiPSC-CFs) and cardiomyocytes (hiPSC-CMs) were subjected to hypoxic conditions. Intriguingly, we observed a significant upregulation of H19 expression in hiPSC-CFs under hypoxic conditions (Figure 3F). Surprisingly, we observed a significant upregulation of H19 in hiPSC-CFs instead of hiPSC-CMs under hypoxic conditions (Figure S9). We then examined the NIH3T3 cells (mouse fibroblasts) under similar conditions, and similar results were observed (Figure 3G).These findings demonstrate that H19 is predominantly expressed in cardiac fibroblasts and excited under hypoxic conditions. Under hypoxic conditions, the upregulation of hypoxia-inducible factors (HIFs), especially HIF-1α, is the primary transcriptional response to hypoxic stress 49. We further examined the regulatory function of HIF-1α on H19. Interestingly, knockdown of HIF-1α significantly downregulated H19 expression in both normoxic and hypoxic conditions (Figure S10), suggesting HIF-1α is an upstream regulatory factor of H19.

To elucidate the mechanism of lncRNA H19 in affecting the changes in fibrosis, we performed chromatin isolation by RNA purification (ChIRP) in NIH3T3 cells to capture RNA binding proteins that interact with lncRNA H19 and conducted mass spectrometry analysis (Figure 3H). Based on the results, there were several interesting candidates which caught our attention, especially Y-box-binding protein 1 (YB-1) (Figure 3I and Table S2). YB-1, belonging to the cold shock protein superfamily, is a DNA- and RNA- binding protein able to regulate gene expression at the transcriptional and translational levels 50. Recently, several studies have revealed that YB-1 is highly correlated with fibrosis 51, 52. Induced-nucleus translocation of YB-1 using a small compound HSc025 showed improvement in hepatic fibrosis as the collagen expression is suppressed 52. In addition, YB-1 was demonstrated to attenuate fibrosis through direct binding onto the Col1a1 promoter in a renal fibrosis model 51. Collectively, YB-1 is important to modulate fibrosis. Nevertheless, the functional role of YB-1 in the heart is still unexplored. Here the H19-YB-1 interaction was confirmed by immunoblotting of YB-1 in ChIRP of H19 (Figure 3J), and H19 was detected by RNA immunoprecipitation (RIP) of YB-1 protein in different sources of cardiac fibroblasts (Figure 3K). Interestingly, lncRNA H19 was found to associate with YB-1 especially under hypoxic conditions (Figure 3K). To assess whether lncRNA H19 and YB-1 interact directly, RNA electrophoretic mobility shift assay (EMSA) was performed. An RNA sequence motif of YB-1, UCCAG/ACAA, was identified in a previous study 53. We found a similar sequence motif located in 661-745 bp of lncRNA H19 and demonstrated that YB-1 indeed binds to lncRNA H19 directly (Figure 3L) with a dissociation constant (K_d_) 7.4 µM (Figure 3M). Since YB-1 is a transcription factor and is able to translocate into the nucleus under hypoxia 54, we examined the localization of YB-1 and H19, respectively. Immunofluorescence analysis of YB-1 revealed that over 70% of cells have YB-1 localized into the nucleus under hypoxic conditions in NIH3T3 cells (Figure 3N). *In vivo*, we also noticed that the nucleus translocation of YB-1 was perceived in cardiac fibroblasts at the infarcted area on D4 post-MI (Figure 3O-P). Besides, we observed that lncRNA H19 was significantly increased in the nucleus under hypoxia (Figure 3Q). Strikingly, the H19-YB-1 interaction was observed in the nucleus under hypoxia (Figure 3R), which implies that the functional mechanism of H19 and YB-1 occurs in the nucleus.

### Knockdown of YB-1 exacerbates cardiac dilation and fibrosis

**Figure 4:**
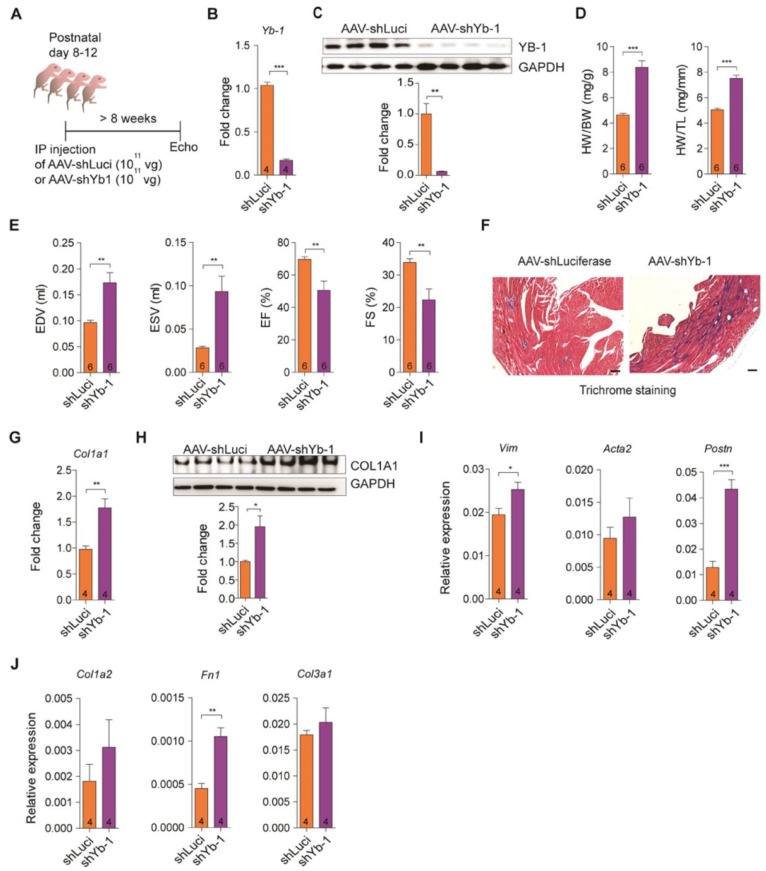
** Knockdown of YB-1 exacerbates cardiac dilation and fibrosis. (A)** Diagram of the experimental design. A single dose of 1 x 10^11^ vg of AAV9-shLuci or AAV-shYB-1 was injected into mice at postnatal day 8-12. Echocardiography was performed on 8-week-old mice post-injection.** (B-C)** Relative (B) Yb-1 expression and (C) immunoblotting of YB-1 on 8-week-old mice. Data represent means ± SEM, **P < 0.01, ***P < 0.001, Student's t-test. **(D)** Heart weight to body weight and heart weight to tibia length ratios on both AAV-shLuci and AAV-shYB-1 injected mice. Data represent means ± SEM, ***P < 0.001, Student's t-test.** (E)** Echocardiographic analysis of mice at 8 weeks post-injection. Data represent means ± SEM, **P < 0.01, Student's t-test.** (F)** Representative images of Trichrome staining of whole hearts, scale bar: 200 µm. **(G-H)** Relative (G) Col1a1 expression and (H) immunoblotting of COL1A1 after AAV injection. Data represent means ± SEM, *P < 0.05, **P < 0.01, Student's t-test.** (I-J)** Relative expression of (I) fibrosis markers Acta2, Postn and Vim, and (J) extracellular matrix components Col1a2, Col3a1 and Fn1. Data are expressed as mean ± SEM, *P < 0.05, **P < 0.01, ***P < 0.001, Student's t-test.

YB-1 has been identified as a repressor of the COL1A1 gene 55. Several studies have pointed out that YB-1 is crucial in regulating collagen expression 51, 52. Nevertheless, the role of YB-1 in the heart remains unexplored. To examine the YB-1 function *in vivo*, we injected a single dose of 1 × 10^11^ viral genome of AAV-shluciferase and AAV-shYB-1 intraperitoneally into mice at postnatal day 8-12, to knockdown YB-1 in mouse hearts (Figure 4A). The YB-1 knockdown efficiency was confirmed (Figure 4B-C). Intriguingly, after YB-1 knockdown the mice showed significantly increased ratios of the heart weight-to-body weight and of the heart weight-to-tibia length (Figure 4D). Moreover, the EDV and ESV increased significantly while the EF and FS decreased in YB-1 knockdown mice (Figure 4E), indicating a pathological remodeling of the heart. Severe fibrosis was also observed in YB-1 knockdown mice (Figure 4F). Since YB-1 regulates Col1a1 expression 55, we examined the Col1a1 expression in YB-1 knockdown mice, and found that the Col1a1 expression was significantly increased both at the mRNA and protein levels (Figure 4G-H). Interestingly, the fibrosis markers (Vim, Acta2 and Postn) and ECM genes (Col1a2, Col3a1 and Fn1) were upregulated in YB-1 knockdown mice (Figure 4I-J), demonstrating that knockdown of YB-1 promotes cardiac fibrosis and ECM remodeling.

### YB-1 transcriptionally regulates Col1a1 expression in cardiac fibroblasts

**Figure 5:**
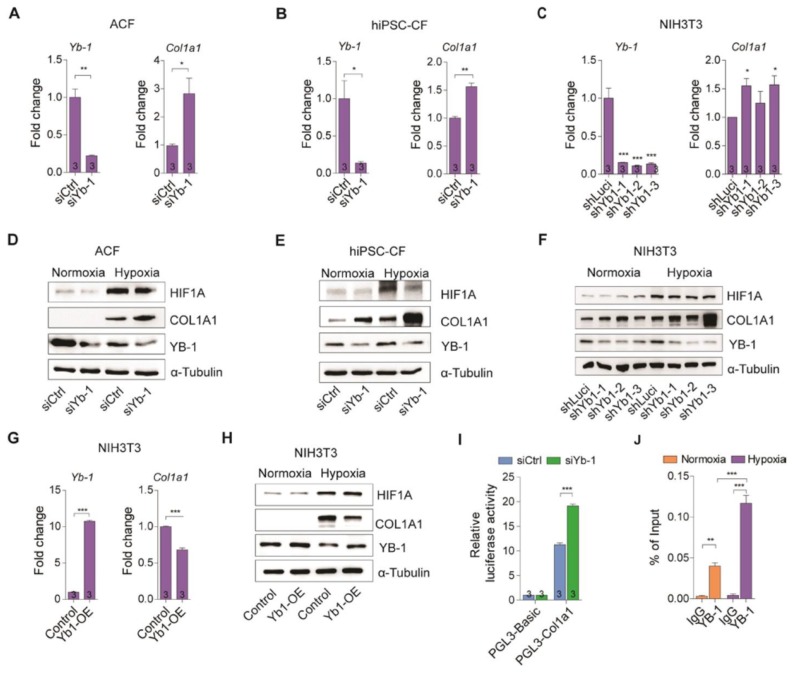
** YB-1 transcriptionally regulates Col1a1 expression. (A-C)** Yb-1 and Col1a1 expression in (A) adult cardiac fibroblasts (ACF), (B) human iPSC-derived cardiac fibroblasts (hiPSC-CF) and (C) NIH3T3 cells under a hypoxic condition after knockdown of Yb-1. Data represent means ± SEM, *P < 0.05, **P < 0.01, ***P < 0.001, Student's t-test. **(D-F)** Representative images of immunoblotting of YB-1 and COL1A1 after knockdown of YB-1 in (D) adult cardiac fibroblasts (ACF), (E) human iPSC-derived cardiac fibroblasts (hiPSC-CF) and (F) NIH3T3 cells under normoxic and hypoxic conditions. **(G)** Yb-1 and Col1a1 expression in NIH3T3 cells under hypoxia after overexpression of Yb-1. Data represent means ± SEM, ***P < 0.001, Student's t-test. **(H)** Representative images of immunoblotting of YB-1 and COL1A1 after overexpression of YB-1 in normoxic and hypoxic conditions. **(I)** Col1a1 promoter assay in the presence and absence of YB-1. Data represent means ± SEM, ***P < 0.001, one-way ANOVA. (J) Chromatin immunoprecipitation (ChIP) of YB-1 under normoxic and hypoxic conditions to detect binding to the Col1a1 promoter. Data represent means ± SEM, **P < 0.01, ***P < 0.001, one-way ANOVA.

We have shown above that lncRNA H19 interacts directly with YB-1 protein and both lncRNA H19 and YB-1 are highly correlated with Col1a1 (Figure 3). We next assessed the functional mechanism of YB-1 in regulation of Col1a1 expression *in vitro*. As expected, knockdown of YB-1 significantly increased the mRNA and protein levels of COL1A1 under hypoxia in adult cardiac fibroblasts, hiPSC-CFs and NIH3T3 cells (Figure 5A-F and Figure S11A-C). Conversely, overexpression of YB-1 in NIH3T3 cells dramatically decreased COL1A1 under hypoxic conditions (Figure 5G-H and Fig S11D). To assess their functionality, we performed promoter assay in the presence and absence of YB-1, respectively. As shown in Figure 5I, the luciferase activity was increased significantly in YB-1 deficiency, confirming that YB-1 is a repressor of Col1a1. Next, we performed chromatin immunoprecipitation (ChIP) to determine whether YB-1 was recruited to the Col1a1 promoter region. Indeed, the recruitment of YB-1 to the Col1a1 promoter was enhanced, especially under hypoxia (Figure 5J). Collectively, these findings suggest that YB-1 acts as a transcriptional suppressor of Col1a1 under hypoxia.

### H19 negatively regulates YB-1 function in Col1a1

**Figure 6:**
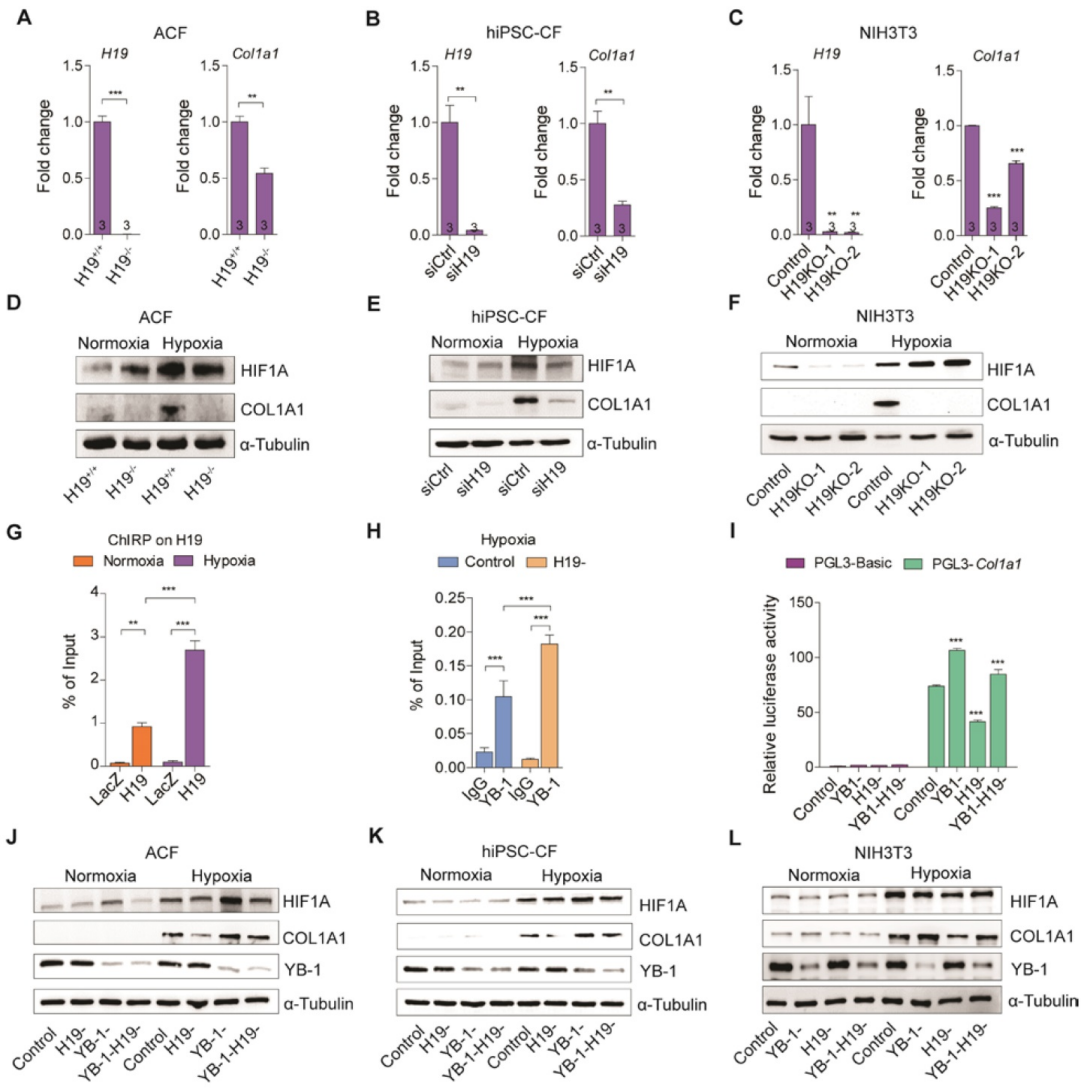
** H19 negatively regulates YB-1 function in controlling Col1a1 expression. (A-C)** H19 and Col1a1 expression in (A) adult cardiac fibroblasts (ACF) isolated from H19 knockout and wild-type mice, (B) human iPSC-derived cardiac fibroblasts (hiPSC-CF) after knockdown of H19 using siRNAs and (C) NIH3T3 cells after knockout of H19 using the CRISPR-Cas9-mediated genome editing approach under a hypoxic conditions. Data represent means ± SEM, **P < 0.01, ***P < 0.001, Student's t-test. **(D-F)** Representative images of immunoblotting for COL1A1 in (D) adult cardiac fibroblast (ACF), (E) human iPSC-derived cardiac fibroblasts (hiPSC-CF) and (F) NIH3T3 cells under normoxic and hypoxic conditions. **(G)** Col1a1 promoter was detected after pull downed of H19 through ChIRP under nomoxia and hypoxia. Data represent means ± SEM, **P < 0.01, ***P < 0.001, one-way ANOVA. **(H)** Chromatin immunoprecipitation (ChIP) of YB-1 in the absence of H19 under hypoxia. Data represent means ± SEM, ***P < 0.001, one-way ANOVA. **(I)** Col1a1 promoter assay in the absence of Yb-1, H19 or both. Data represent means ± SEM, ***P < 0.001, one-way ANOVA. **(J-L)** Representative images of immunoblotting for COL1A1 in the absence of YB-1, H19 and both in (J) adult cardiac fibroblasts (ACF), (K) human iPSC-derived cardiac fibroblasts (hiPSC-CF) and (L) NIH3T3 cells under normoxia and hypoxia.

The results presented above drove us to hypothesize that lncRNA H19 and YB-1 are interrelated in the regulation of COL1A1 expression. To test this hypothesis, we first evaluated COL1A1 expression in adult cardiac fibroblasts, hiPSC-CFs and NIH3T3 cells. Interestingly, the expression of COL1A1 mRNA and protein were significantly decreased in the absence of H19 (Figure 6A-F and Figure S12A-C). While, an opposite result was observed with H19 overexpression in NIH3T3 cells (Figure S12D-F). To further assess whether the knockout of H19 affects COL1A1 secretion, immunofluorescence and immunoblotting analysis were performed on total secreted ECM to detect the presence of COL1A1. As shown in Figure S12G, the secreted COL1A1 decreased significantly with H19 knockout in NIH3T3 cells. To examine the functionality of lncRNA H19, cell proliferation and apoptosis assay were performed *in vivo* (Figure S13A-B) and *in vitro* (Figure S13C-F), respectively. However, no significant differences were observed. To examine the interrelationship between H19 and YB-1, we performed ChIRP to determine whether Col1a1 promoter is one of the lncRNA H19 targeted loci. Surprisingly, Col1a1 promoter was highly detected especially under hypoxic conditions (Figure 6G). We also performed chromatin immunoprecipitation (ChIP) to evaluate the recruitment of YB-1 to Col1a1 promoter in the absence of H19 under hypoxia condition. Intriguingly, the recruitment of YB-1 to the Col1a1 promoter was exceptionally elevated without the existence of lncRNA H19 (Figure 6H), suggesting that lncRNA H19 disrupts the recruitment of YB-1 to the Col1a1 promoter by direct binding to YB-1 protein. To further evaluate the functional assessment, we performed a promoter assay in the absence of YB-1, H19 or both. As shown in Figure 6I, the luciferase activity was increased significantly in YB-1 deficiency but decreased exceptionally without the existence of H19. Notably, luciferase activity bounced back to normal as both H19 and YB-1 were absent. A similar trend was observed when COL1A1 was immunoblotted in the absence of YB-1, H19 or both in adult cardiac fibroblast, hiPSC-CFs and NIH3T3 cells (Figure 6J-L). These findings suggest that H19 is a fibrosis regulator during the cardiac remodeling process after infarction.

## Discussion

Unlike the wound healing process in other tissues, activated cardiac fibroblasts in injured hearts persist and continue to synthesize ECM proteins 48. Although the ECM is able to sustain myocardium structural integrity, excessive accumulation of ECM can be detrimental to survival 4, 5. To date, different therapies targeting the ECM in human patients with myocardial fibrosis have resulted in improvement in cardiac functional parameters although the effects have been relatively modest due to the non-targeted nature of these drugs 56-59. Therefore, development of specific therapies targeting the ECM has become a priority. In this study, we unraveled the crucial role of an interaction between H19-YB-1 in regulating cardiac fibrosis after infarction and demonstrated that lncRNA H19 is an ECM regulator which may serve as a therapeutic target for ischemic cardiomyopathy.

LncRNAs have become powerful therapeutic targets due to their diverse functions at the cellular level 60. They have been studied intensively both with respect to heart development 61, 62 and cardiac disease progression 63, 64. Several studies have shown that lncRNAs regulate cardiac fibrosis through different mechanisms such as transcriptional modification and regulation 40, 65 and serve as a microRNA sponge to inhibit microRNA function 63, 66. Our data revealed that lncRNA H19 regulates COL1A1 expression at the early stage of cardiac remodeling. Our detailed mechanistic study showed that lncRNA H19 acts as a molecular decoy that interacts and titrates away YB-1 protein, a DNA- and RNA- binding protein that serves as suppressor of COL1A1. Our study provides new functional mechanistic insight into lncRNA H19 in ECM regulation.

We noticed the re-expression of lncRNA H19 was only observed in the MI-induced heart failure model suggesting that the depletion of oxygen in the MI model might promote lncRNA H19 expression. In glioblastoma, H19 was upregulated under hypoxic stress due to the induction of transcriptional activator specific protein 1 (SP1), which was induced by hypoxia-inducible factor 1α (HIF-1α) under hypoxia 67, 68. This may provide a potential upstream regulatory mechanism for H19 re-expression in ischemic heart diseases. Hypoxia not only provides a potential regulator of gene expression but also triggers YB-1 nuclear localization. Such a micro-environmental cue in the hypoxic myocardium is crucial for the interaction of H19 and YB-1 for the regulation of Collagen 1A1 transcription. Indeed, the nucleus translocation of YB-1 is important as reported in several studies where the nucleus translocation of YB-1 is able to reduce fibrosis in the kidneys and liver 51, 52.

The functions of H19 are diverse, one of them is to serve as a reservoir for miR-675 69. Since miR-675 is a microRNA, it is involved in a diverse array of signaling pathways by targeting a myriad of transcripts 69-72. Hence, H19 is able to regulate a wide-range of biological processes via miR-675. Besides, the full-length of H19 transcript provides a stable secondary structure which serves as an interacting platform for diverse proteins. Therefore, H19 can exert its function through interacting with proteins 73-75. Another function of H19 is to serve as a “sponge” to absorb miRNAs and disable their functions 76, 77. The role of H19 has been previously mentioned in cardiomyopathy studies, but these studies mainly focus on H19-encoded microRNA, miR-675 78, 79. For example, lncRNA H19 was demonstrated to regulate cardiac hypertrophy and cardiomyocyte apoptosis through inhibition of CaMKIIδ and VDAC1 expression by miR-675 encoded in exon 1 of H19 78, 79. The role of lncRNA H19 itself in cardiac pathogenesis is not clearly defined. In our experiment, we directly pulled-down H19 and identified YB-1 as an interacting protein of lncRNA H19. We observed severe fibrosis in the heart after knockdown of YB-1 using shRNAs *in vivo*. Surprisingly, the phenotypic expression of YB-1 deficiency mice was similar to H19OE mice with infarction. Other interesting proteins were identified by mass spectrometry using ChIRP to pull down lncRNA H19 including ANXA2, DESP, DSG1A and FBXL4 which are involved in fibrin homeostasis, cell-cell adhesion, junction assembly and mtDNA maintenance, respectively. Defects in these proteins are linked to several different cardiac diseases 80-82; nevertheless, the functional roles of these proteins relative to the development of fibrosis in the heart remains unclear.

LncRNA H19 is highly abundant and extensively upregulated in cardiac fibroblasts after injury (Figure 3). Unlike Wisper 40, modulation of lncRNA H19 did not show significant changes in cardiac fibroblast proliferation or apoptosis (Figure S13). However, modulation of lncRNA H19 expression *in vivo* (Figure 1 and 2) led to transcriptional changes in the ECM components, suggesting that H19 regulates ECM expression. In addition, collagens and fibronectin, and other ECM components secreted after injury 83, are regulated, which suggests that lncRNA H19 is a crucial regulatory factor of ECM components. In our study, we identified YB-1 as an H19 interacting protein that specifically regulates COL1A1 expression, likely through formation of the H19-YB-1-complex and their nuclear translocation. Besides, we also noticed other ECM components like Col1a2, Col3a1 and Fn1 had upregulated expression in YB-1 knockdown mice. A study demonstrated that YB-1 mediates the inhibitory action on Col1a2 84. We further assessed whether YB-1 transcriptionally modulates the expression of these genes through promoter assay after knockdown of YB-1 in NIH3T3 cells. Surprisingly, luciferase activities in these three genes were significantly upregulated in NIH3T3 cells after knockdown of YB-1, suggesting that YB-1 is the transcriptional suppressor for Col1a2, Col3a1 and Fn1 (Figure S14).

While we have not omitted the possibility of miR-675 contribution to cardiac fibrosis, when lncRNA H19 was knocked-out, miR-675 was barely detected (Figure S15A); once H19 was expressed, miR-675 was expressed concurrently (Figure S15B). However, our experimental design omitted investigation of the involvement of miR-675 in this study. Further, H19 was discovered as a highly conserved imprinted gene cluster together with nearby reciprocally imprinted gene, insulin-like growth factor 2 (Igf2) 85. We examined the Igf2 expression in both H19OE and H19KO mice after injury to assess whether modulation of H19 affects Igf2 expression. We noticed no significant differences of the Igf2 expression in either groups (Figure S15C-D), suggesting that the function of H19 may be independent of Igf2.

According to our hypothetical model, Co1a1 is upregulated after MI at the same time as nuclear translocation of the negative regulator YB-1. As a transcriptional repressor, YB-1 suppresses Col1a1 expression even when endogenous Col1a1 is induced. When ischemia continues, lncRNA H19 is slowly upregulated and reaches an exceptionally high level at post-MI D4. Regarding the binding affinity of H19 and YB-1, YB-1 may prefer to interact with lncRNA H19 compared to the COL1A1 promoter when lncRNA H19 is present. LncRNA H19 then competes with COL1A1 promoter to form the H19-YB-1 complex. When the H19-YB-1 complex is established, the function of YB-1 as a suppressor of COL1A1 is abolished and the expression of Col1a1 is increased.

In summary, we have revealed a new mechanistic pathway for cardiac remodeling through H19-YB-1 interaction. Inhibition of H19 expression or the interaction of H19 and YB-1 by oligonucleotides at the early stage of cardiac remodeling may provide a novel therapeutic strategy whereby the function of YB-1 as a suppressor of COL1A1 expression is conserved thus preventing ECM deposition and cardiac remodeling after heart injury.

## Abbreviations

lncRNA: long noncoding RNA
MI: myocardial infarction
ECM: extracellular matrix
KO: knockout
OE: overexpression
AAV: adeno-associated virus
ChIRP: chromatin isolation by RNA purification
