# Supplementary material for: Hypoxia-induced H19/YB-1 cascade modulates cardiac remodeling after infarction
Source: Theranostics. 2019 Aug 21;9(22):6550–67. doi: 10.7150/thno.35218 (PMC6771230; doi:10.7150/thno.35218)
Supplement: Supplementary file 1 — Supplementary figures and tables. [file thnov09p6550s1.pdf]

SUPPLEMENTARY

Hypoxia-induced H19/YB-1 cascade modulates cardiac remodeling after infarction

Oi Kuan Choong, Chen-Yun Chen, Jianhua Zhang, Jen-Hao Lin, Po-Ju Lin, Shu-Chian Ruan, Timothy J. Kamp, Patrick C.H. Hsieh

Supplementary figures 1-15

Supplementary tables 1-5

Figure S1

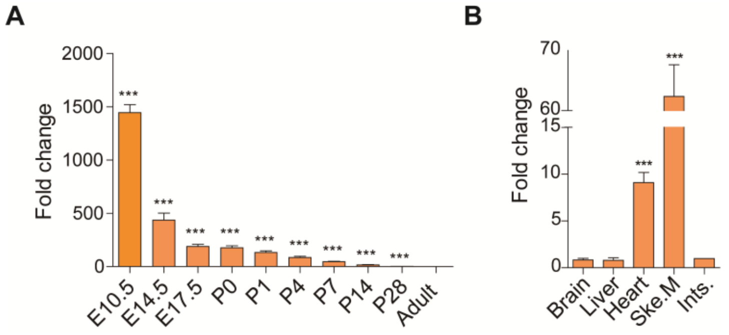

Figure S1. Expression of lncRNA H19 in the heart.

A H19 expression during mouse heart development from embryonic day 10.5 (E10.5) until adulthood (n=8). Data represent means ± SEM, ****P* < 0.001, one-way ANOVA.

B H19 expression in adult organs including the brain, liver, heart, skeletal muscle (Ske.M) and intestine (Ints.) (n=7). Data represent means ± SEM, ****P* < 0.001, one-way ANOVA.

Figure S2

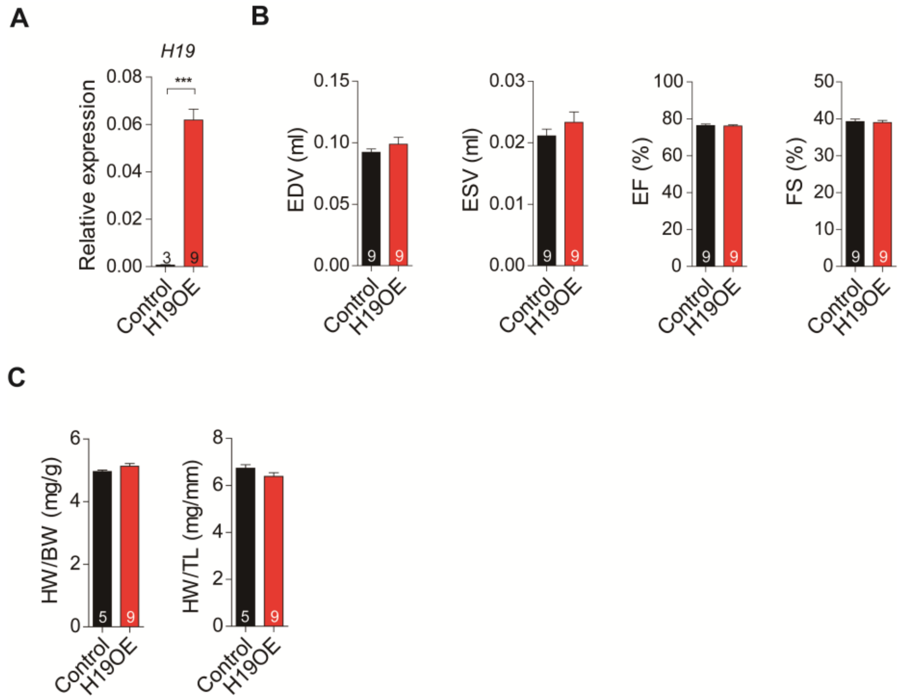

Figure S2. Baseline characterization of H19 overexpression mice.

A Expression of H19 in control and H19 overexpression (H19OE) mice prior to injury. Data represent means ± SEM, ****P* < 0.001, Student's t-test.

B Echocardiography analysis prior to MI, EDV (end diastolic volume), ESV (end systolic volume), EF (ejection fraction) and FS (fraction shortening), for control and H19OE mice.

C Heart weight to body weight and heart weight to tibia length ratios in control and H19OE mice prior to MI.

Figure S3

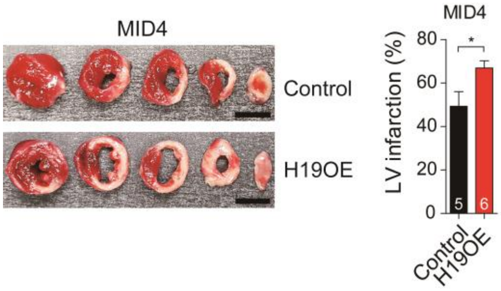

Figure S3. Triphenyltetrazolium chloride (TTC) staining on H19 overexpression mice after injury.

Representative images for TTC staining of the whole heart after MI in both control and H19 overexpression (H19OE) groups, scale bar: 5 mm. Data are expressed as mean ± SEM, *P < 0.05, Student's t-test.

Figure S4

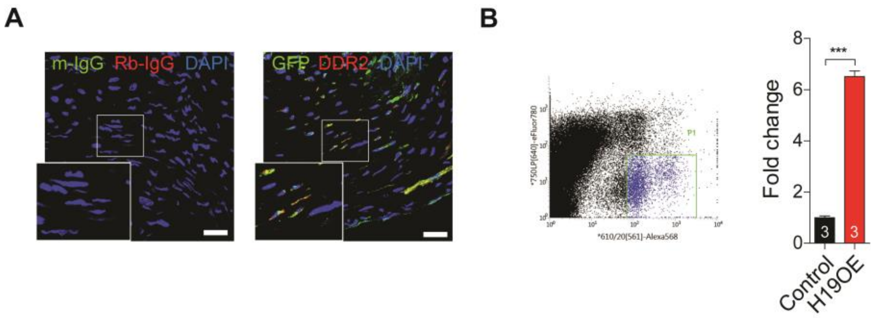

Figure S4. Detection of GFP in cardiac fibroblasts post-AAV injection.

A GFP was detected in DDR2^{+} cardiac fibroblasts at the infarcted area in the AAV9-injected mouse heart, scale bar: 20 μm.

B PDGFR-α^{+} cells were sorted and evaluated for H19 expression. Data represent means ± SEM, ***$P < 0.001$, Student’s t-test.

Figure S5

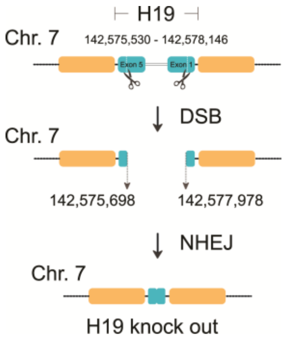

Figure S5. Generation of H19 knockout mice.

Diagram showing generation of H19 knockout mice using CRISPR-Cas9-mediated genome editing.

Figure S6

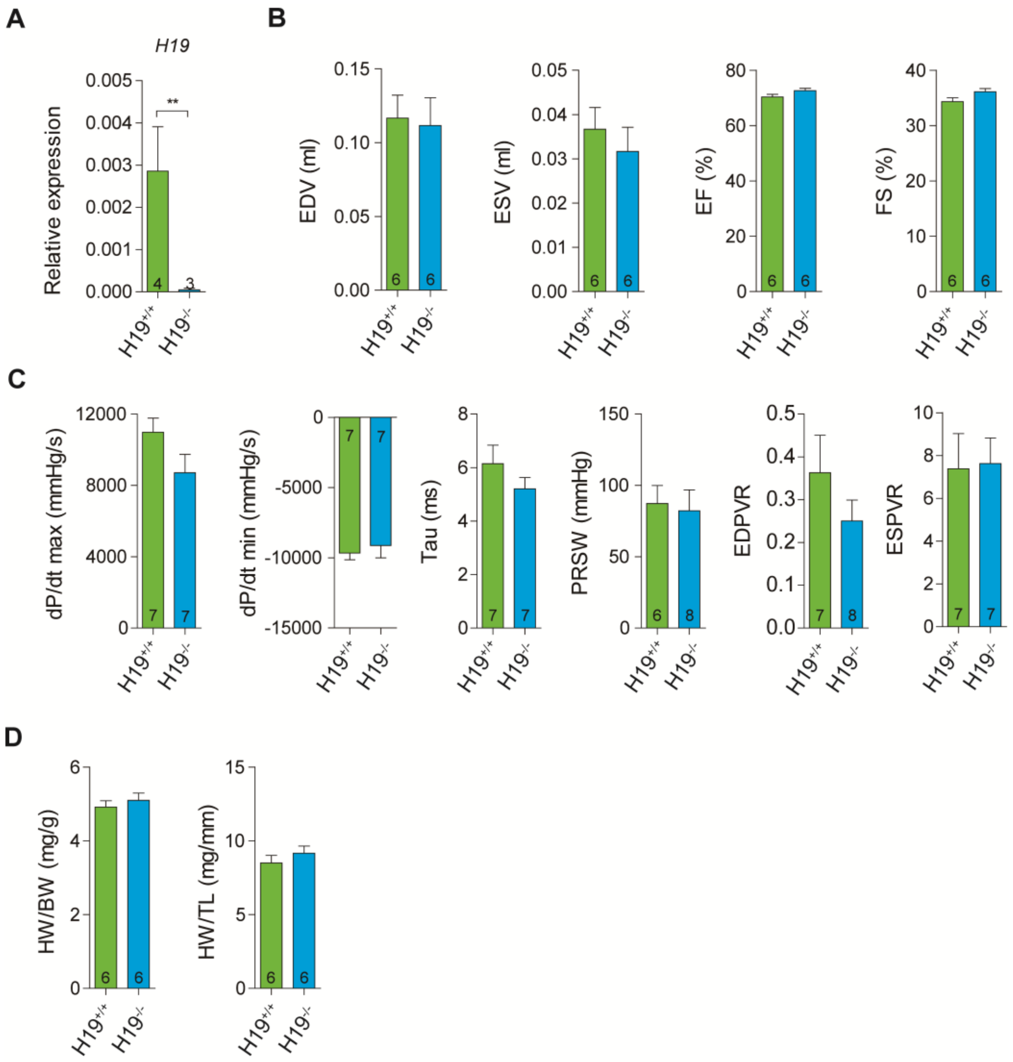

Figure S6. Baseline characterization of H19 knockout mice.

A Expression of H19 in wild-type ($H19^{+/+}$) and homozygous H19 knockout ($H19^{-/-}$) mice prior to injury. Data represent means ± SEM, $**P < 0.01$, Student's t-test.

B Echocardiography analysis prior to MI, EDV (end diastolic volume), ESV (end systolic volume), EF (ejection fraction) and FS (fraction shortening), for $H19^{+/+}$ and $H19^{-/-}$ mice.

C Cardiac catheterization analysis for $H19^{+/+}$ and $H19^{-/-}$ mice. Peak rate of pressure rise (dP/dt_{max}), preload recruited stroke work (PRSW), end-systolic pressure–volume relation (ESPVR), peak rate of pressure decline (dP/dt_{min}), relaxation time constant (Tau), end-diastolic PV relation slope (EDPVR) were evaluated.

D Heart weight to body weight and heart weight to tibia length ratios in $H19^{+/+}$ and $H19^{-/-}$ mice prior to MI.

Figure S7

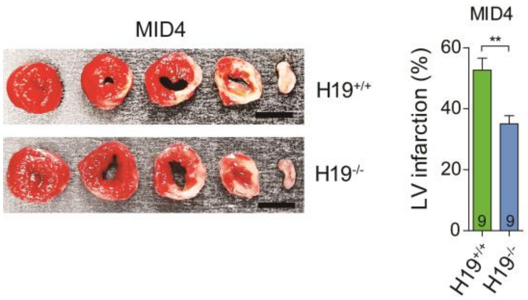

Figure S7. Triphenyltetrazolium chloride (TTC) staining on H19 knockout mice after injury. Representative images for TTC staining of the whole heart after MI in both wild type (H19$^{+/+}$) and H19 knockout (H19$^{-/-}$) groups, scale bar: 5 mm. Data are expressed as mean ± SEM, $**P < 0.01$, Student's t-test.

Figure S8

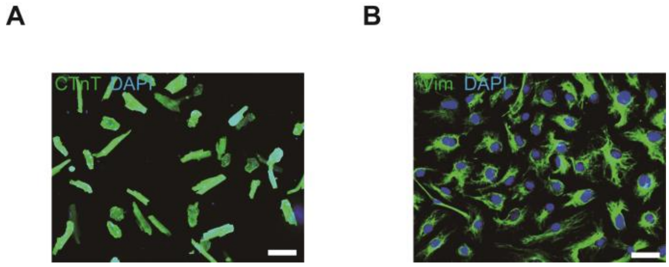

Figure S8. Isolated mouse adult cardiomyocytes and cardiac fibroblasts.

A Representative images of immunostaining of isolated mouse adult cardiomyocytes, CTnT (green), nucleus (blue), scale bar: 100 µm.

B Representative images of immunostaining of isolated mouse cardiac fibroblast, Vim (green), nucleus (blue), scale bar: 50 µm.

Figure S9

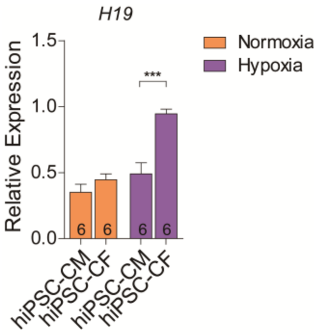

Figure S9. H19 gene expression under normoxic and hypoxic conditions.

Comparison of H19 gene expression in human iPSC-derived cardiomyocytes (hiPSC-CM) and human iPSC-derived cardiac fibroblasts (hiPSC-CF) under normoxic and hypoxic conditions. Data represent means ± SEM, ****P* < 0.001, one-way ANOVA.

Figure S10

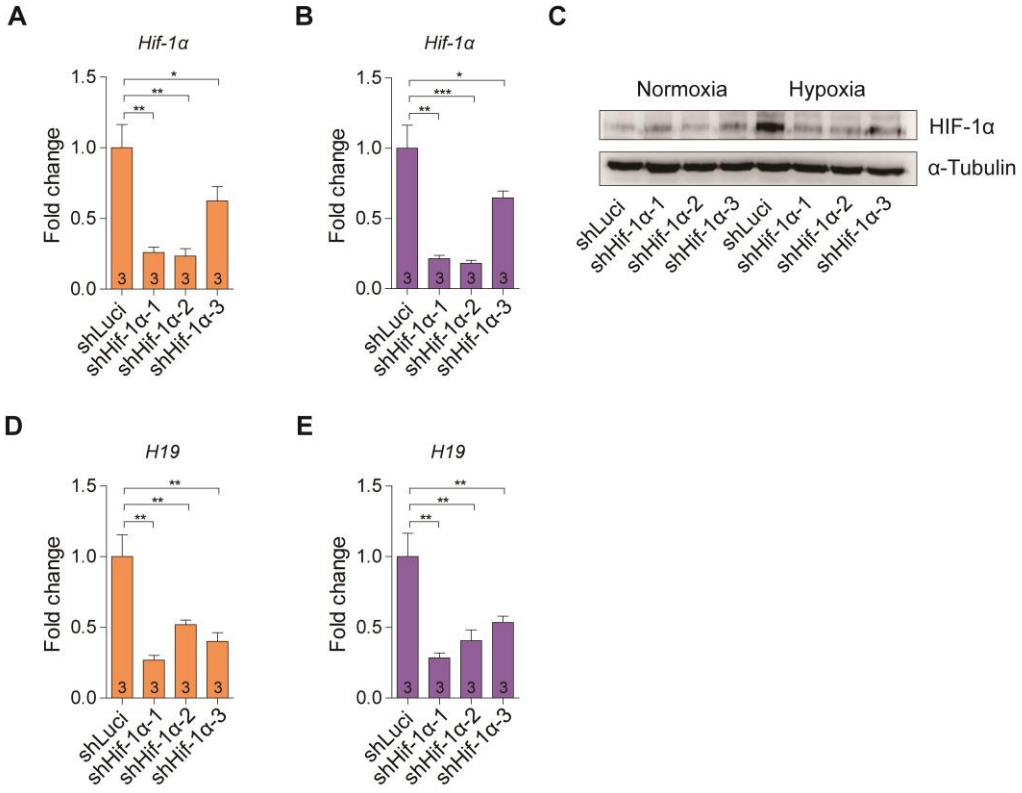

Figure S10. Knockdown of Hif-1α downregulates H19 expression.

A,B The expression of Hif-1α after knockdown of Hif-1α using shRNAs in NIH3T3 cells under (A) normoxic and (B) hypoxia conditions. Data are shown as mean ± SEM, $*P < 0.05$, $**P < 0.01$, $***P < 0.001$, one-way ANOVA.

C Representative images for immunoblotting of HIF-1α after knockdown of Hif-1α in NIH3T3 cells under normoxic and hypoxic conditions.

D,E H19 expression after knockdown of Hif-1α using shRNAs in NIH3T3 cells under (A) normoxic and (B) hypoxia conditions. Data are shown as mean ± SEM, $**P < 0.01$, one-way ANOVA.

Figure S11

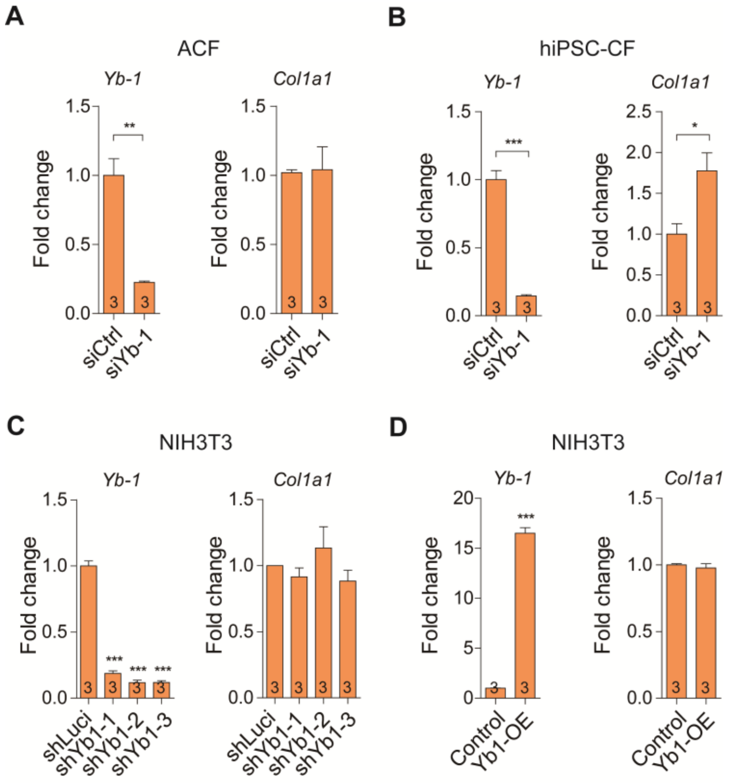

Figure S11. Yb-1 and Col1a1 gene expressions under normoxia condition.

A,B Yb-1 and Col1a1 expressions in normoxic condition after knockdown of Yb-1 using siRNA in (A) mouse adult cardiac fibroblasts and (B) human iPSC-derived cardiac fibroblasts. Data represent means ± SEM, *$P < 0.05$, **$P < 0.01$, ***$P < 0.001$, Student's t-test.

C Yb-1 and Col1a1 expressions in normoxic condition after knockdown of Yb-1 using shRNAs in NIH3T3 cells. Data represent means ± SEM, ***$P < 0.001$, one-way ANOVA.

D Yb-1 and Col1a1 expressions in normoxic condition after Yb-1 overexpression (Yb1-OE) in NIH3T3 cells. Data represent means ± SEM, ***$P < 0.001$, Student's t-test.

Figure S12

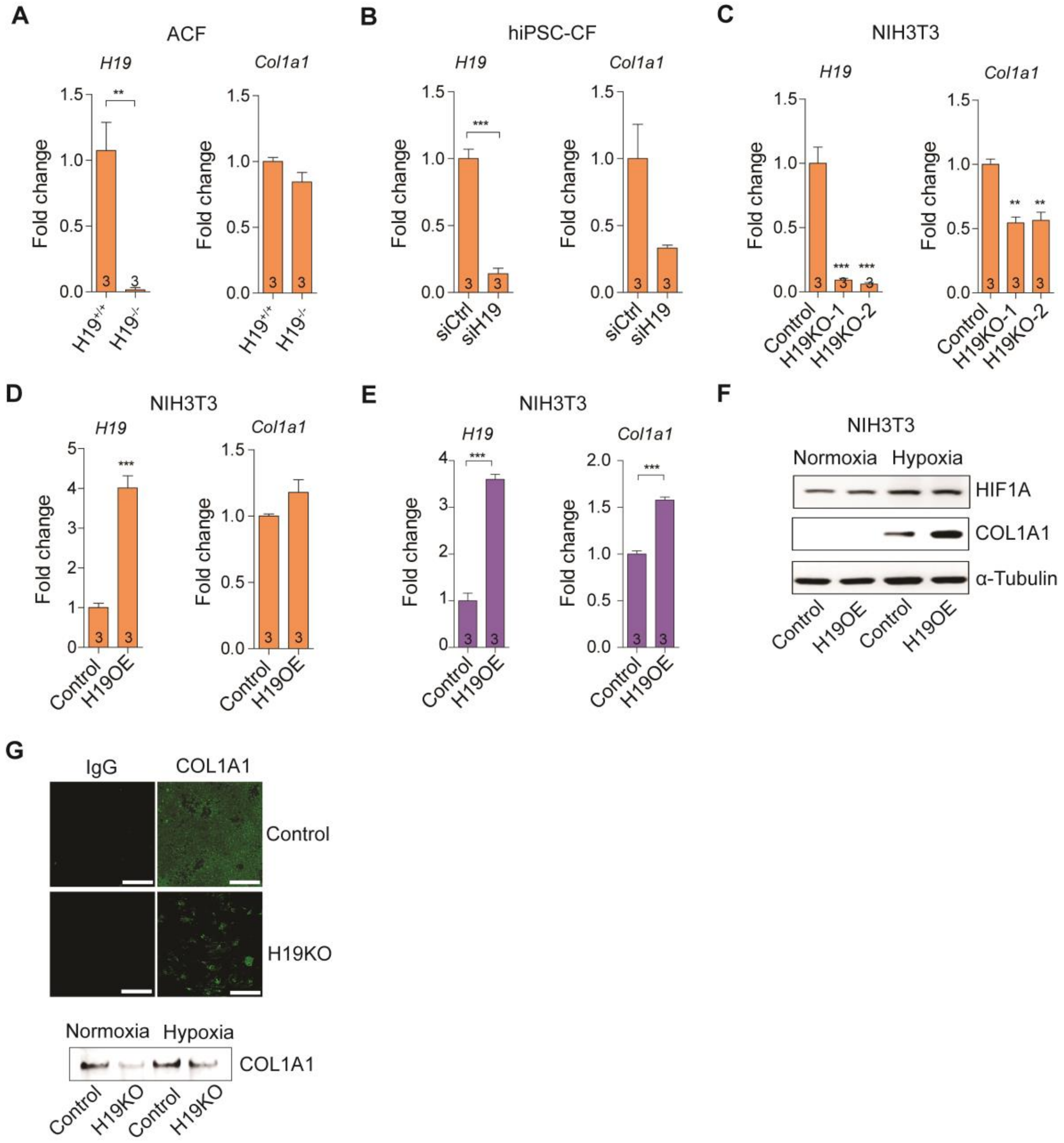

Figure S12. H19 and Col1a1 gene expression under normoxia or hypoxia.

A H19 and Col1a1 expression under normoxic conditions in mouse adult cardiac fibroblasts from $H19^{+/+}$ and $H19^{-/-}$ mice. Data represent means ± SEM, $**P < 0.01$, Student's t-test.

B H19 and Col1a1 expressions under normoxic conditions after knockdown of H19 using siRNA in human iPSC-derived cardiac fibroblasts. Data represent means ± SEM, $***P < 0.001$, Student's t-test.

C,D H19 and Col1a1 expressions under normoxic conditions after (C) knockout of H19 in NIH3T3 cells and (D) overexpressed of H19 (H19OE) in NIH3T3 cells. Data represent means ± SEM, $**P < 0.01$, $***P < 0.001$, one-way ANOVA and Student's t-test, respectively.

E H19 and Col1a1 expressions in NIH3T3 cells under hypoxia after overexpression of H19. Data represent means ± SEM, ****P* < 0.001, Student's t-test.

F Representative images of immunoblotting for COL1A1 after overexpression of H19 under normoxic and hypoxic conditions.

G Representative images of immunofluorescence for COL1A1 in total secreted extracellular matrix *in vitro* and representative images for immunoblotting of COL1A1 in total secreted extracellular matrix, scale bar: 50 μm.

Figure S13

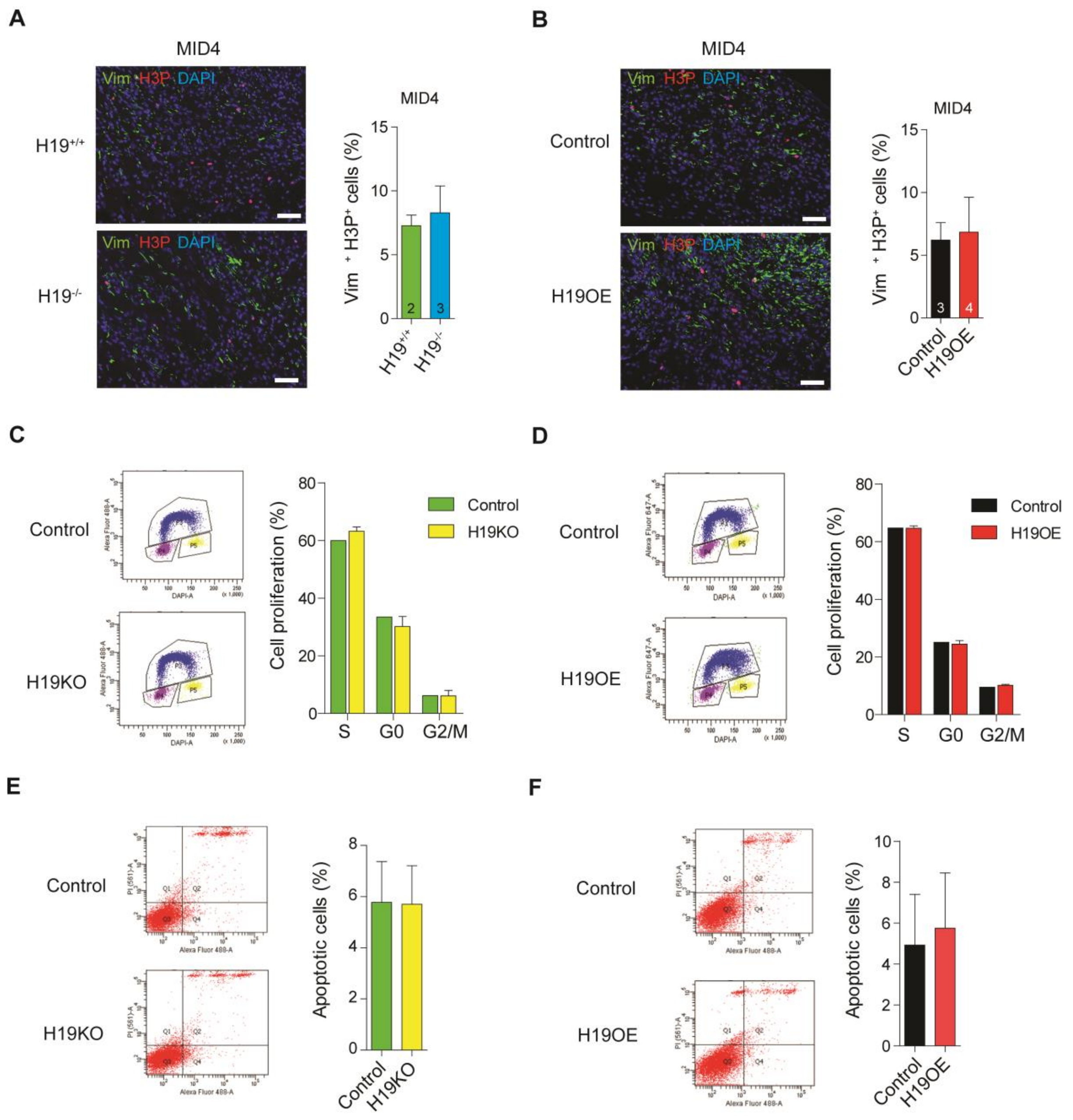

Figure S13. Evaluation of H19 effects in fibroblast proliferation and apoptosis.

A,B Cardiac fibroblast proliferation was evaluated by immunofluorescent staining of fibroblast marker (Vim) and proliferation marker (H3P) in (A) $H19^{+/+}$ and $H19^{-/-}$ mice post-MID4, (B) control and H19OE mice post-MID4. The cell proliferation rate was presented in percentage of double positive cells, scale bar: 100 μm.

C,D Proliferation assay was performed in NIH3T3 cells with (C) H19 knockout and (D) H19 overexpression.

E,F Apoptotic cells were evaluated by flow cytometry through detection of Annexin V in NIH3T3 cells with (E) H19 knockout and (F) H19 overexpression.

Figure S14

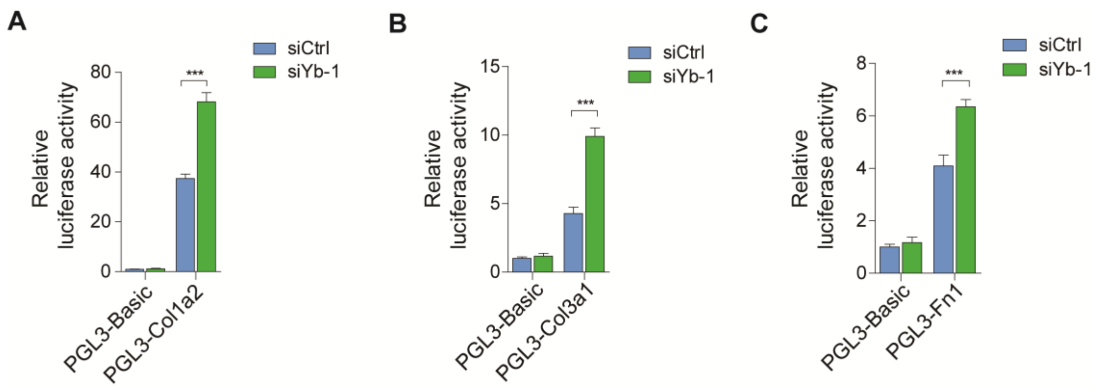

Figure S14. YB-1 is transcriptional suppressor for Col1a2, Col3a1 and Fn1.

A-C (A) Col1a2, (B) Col3a1 and (C) Fn1 promoter assay in the presence and absence of YB-1. Data represent means ± SEM, ****P* < 0.001, one-way ANOVA.

Figure S15

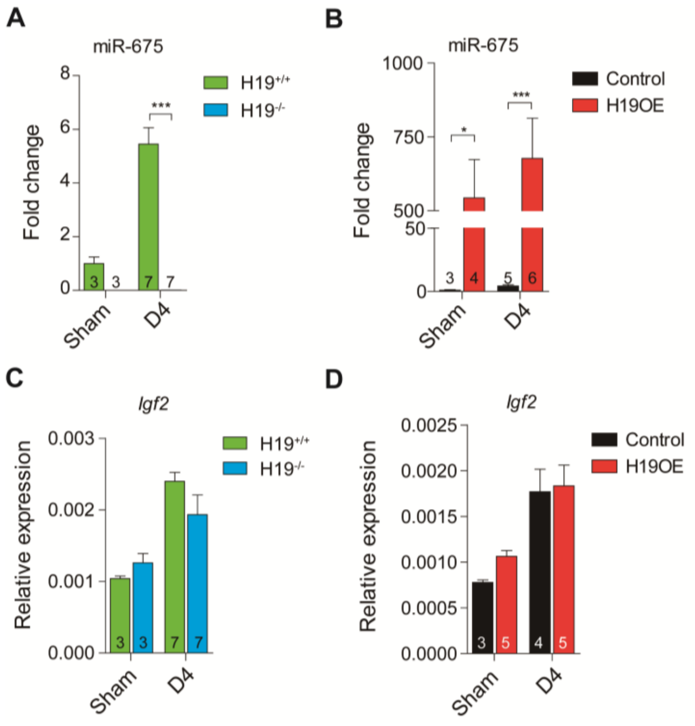

Figure S15. Expression of miR-675 and Igf2 in H19 knockout or overexpression.

A,B Quantification of miR-675 by TaqMan qPCR in mouse hearts with (A) H19 knockout and (B) H19 overexpression in sham or after MI. Data are expressed as mean ± SEM, *$P < 0.05$, ***$P < 0.001$, one-way ANOVA.

C,D qPCR quantification of Igf2 expression in mouse hearts with (C) H19 knockout and (D) H19 overexpression in sham or after MI.

Table S1: Probe sequences for ChIRP

Probes	Sequence
ChIRP-Lacz-1	TTC AAC CAC CGC ACG ATA GA
ChIRP-Lacz-2	CTC GAA TCA GCA ACG GCT TG
ChIRP-Lacz-3	GCG TTA AAG TTG TTC TGC TT
ChIRP-Lacz-4	ATG CCG TGG GTT TCA ATA TT
ChIRP-Lacz-5	GAT CAC ACT CGG GTG ATT AC
ChIRP-Lacz-6	CGC GTA CAT CGG GCA AAT AA
ChIRP-Lacz-7	TAT TCG CAA AGG ATC AGC GG
ChIRP-Lacz-8	TAA TCA GCG ACT GAT CCA CC
ChIRP-Lacz-9	TCG GCA AAG ACC AGA CCG TT
ChIRP-Lacz-10	CGC TAT GAC GGA ACA GGT AT
ChIRP-H19-1	TCA GTC CTT CAA CAT TCC TG
ChIRP-H19-2	CCA CGT CCT GTA ACC AAA AG
ChIRP-H19-3	TAG AAG GTC AGT GGA GCG AG
ChIRP-H19-4	AGA CGA TGT CTC CTT TGC TA
ChIRP-H19-5	CTC AGT CTT TAC TGG CAA CC
ChIRP-H19-6	CAC TCT TGA ACC TTC TTC TA
ChIRP-H19-7	TGT AAA ATC CCT CTG GAG TC
ChIRP-H19-8	ATA CAG TGT ACC AAG TCC AC
ChIRP-H19-9	CTC CCT AGA AAC TCA TTC AT
ChIRP-H19-10	AAT TGA ACT TGC GTG GGA GG
ChIRP-H19-11	TTT CTG TCA CAT TGA CCA CA
ChIRP-H19-12	AAT TAG GTG GTT GAG CGG AC
ChIRP-H19-13	AGA GAG CAG CAG AGA AGT GT
ChIRP-H19-14	TTA AAG AAG TCC CCG GAT TC
ChIRP-H19-15	TTG ACA CCA TCT GTT CTT TC
ChIRP-H19-16	CAG GAT GAT GTG GGT GGT GG
ChIRP-H19-17	ATG GGG AAA CAG AGT CAC GG
ChIRP-H19-18	AAG AGG TTT ACA CAC TCG CT
ChIRP-H19-19	CAG ACT AGG CGA GGG GAA GG
ChIRP-H19-20	ACT GTA TTT ATT GAT GGA CC

Table S2: Mass spectrometry results

No.	Symbols	Protein names
1	YBOX1_MOUSE	Nuclease-sensitive element-binding protein 1
2	ANXA2_MOUSE	Annexin A2
3	UPP_MOUSE	Uracil phosphoribosyltransferase homolog
4	K319L_MOUSE	Dyslexia-associated protein KIAA0319-like protein
5	DESP_MOUSE	Desmoplakin
6	DSG1A_MOUSE	Desmoglein-1-alpha
7	ASAP2_MOUSE	Arf-GAP with SH3 domain, ANK repeat and PH domain-containing protein 2
8	TOP1_MOUSE	DNA topoisomerase 1
9	FBXL4_MOUSE	F-box/LRR-repeat protein 4
10	CASP8_MOUSE	Caspase-8
11	OBSCN_MOUSE	Obscurin
12	MYPT2_MOUSE	Protein phosphatase 1 regulatory subunit 12B
13	TERT_MOUSE	Telomerase reverse transcriptase
14	VIME_MOUSE	Vimentin
15	NEK4_MOUSE	Serine/threonine-protein kinase Nek4
16	DYH12_MOUSE	Dynein heavy chain 12, axonemal
17	INSRR_MOUSE	Insulin receptor-related protein
18	RS27A_MOUSE	Ubiquitin-40S ribosomal protein S27a
19	VASH1_MOUSE	Vasohibin-1
20	QKI_MOUSE	Protein quaking
21	SIK3_MOUSE	Serine/threonine-protein kinase SIK3
22	RPTN_MOUSE	Repetin
23	TTF2_MOUSE	Transcription termination factor 2
24	MARH7_MOUSE	E3 ubiquitin-protein ligase MARCH7
25	AKIB1_MOUSE	Ankyrin repeat and IBR domain-containing protein 1
26	MYH7B_MOUSE	Myosin-7B
27	PUS7L_MOUSE	Pseudouridylate synthase 7 homolog-like protein
28	MEOX2_MOUSE	Homeobox protein MOX-2
29	PTN4_MOUSE	Tyrosine-protein phosphatase non-receptor type 4
30	SEC20_MOUSE	Vesicle transport protein SEC20
31	ATX10_MOUSE	Ataxin-10
32	CNTN5_MOUSE	Contactin-5
33	IFI2_MOUSE	Interferon-activable protein 202

34	U2AFL_MOUSE	U2 small nuclear ribonucleoprotein auxiliary factor 35 kDa subunit-related protein 1
35	CFA69_MOUSE	Cilia- and flagella-associated protein 69

Table S3: Primers for qPCR and ChIP-qPCR

Name	Species	Sequence
**qPCR primers**		
H19-F	Mouse	AAGAGCTCGGACTGGAGACT
H19-R	Mouse	GACCACACCTGTCATCCTCG
YB-1-F	Mouse	GCAGACCGTAACCATTATAGACG
YB-1-R	Mouse	TCTCCGCATGTAGTAAGGTGG
Col1a1-F	Mouse	ACCCGAGGTATGCTTGATCTG
Col1a1-R	Mouse	CATTGCACGTCATCGCACAC
Col1a2-F	Mouse	CCAAGGGTGCTACTGGACTC
Col1a2-R	Mouse	GCTCACCCTTGTTACCGGAT
Postn-F	Mouse	TGCTGCCCTGGCTATATGAG
Postn-R	Mouse	GTAGTGGCTCCCACAATGCC
Vim-F	Mouse	AGACCAGAGATGGACAGGTGA
Vim-R	Mouse	TTGCGCTCCTGAAAAACTGC
Fn1-F	Mouse	ACTGCAGTGACCAACATTGACC
Fn1-R	Mouse	CACCCTGTACCTGGAAACTTGC
Col3a1-F	Mouse	GCCCACAGCCTTCTACAC
Col3a1-R	Mouse	CCAGGGTCACCATTTCTC
Acta2-F	Mouse	GTCCCAGACATCAGGGAGTAA
Acta2-R	Mouse	TCGGATACTTCAGCGTCAGGA
Hprt-F	Mouse	GTT GGG CTT ACC TCA CTG CT
Hprt-R	Mouse	TCA TCG CTA ATC ACG ACG CT
hs-H19-F	Human	TGC TGC ACT TTA CAA CCA CTG
hs-H19-R	Human	ATG GTG TCT TTG ATG TTG GGC
hs-YB1-F	Human	GCG GGG ACA AGA AGG TCA TC
hs-YB1-R	Human	TCC TTG GTG TCA TTC CTG TTG A
hs-COL1A1-F	Human	TGA AGG GAC ACA GAG GTT TCA G
hs-COL1A1-R	Human	GTA GCA CCA TCA TTT CCA CGA
hs-TBP-F	Human	CCA CTC ACA GAC TCT CAC AAC
hs-TBP-R	Human	CTG CGG TAC AAT CCC AGA ACT
**ChIP-qPCR primers**		
COL1A1-pro-F	Mouse	GGATGTCAAAGGTCTCCCCAA
COL1A1-pro-R	Mouse	AGGAAGGGGGTGCCTATCTG

Table S4: Echocardiography data of H19 overexpression mice after MI.

Parameters	Control (MID4)	H19OE (MID4)
No. of mice	8	8
IVSd (mm)	0.342 ± 0.002	0.338 ± 0.003
LVIDd (mm)	4.750 ± 0.015	5.250 ± 0.019*
LVPWd (mm)	0.350 ± 0.002	0.388 ± 0.003
IVSs (mm)	0.542 ± 0.002	0.563 ± 0.002
LVIDs (mm)	3.892 ± 0.014	4.313 ± 0.017
LVPWs (mm)	0.491 ± 0.003	0.538 ± 0.003
SV(ml)	0.113 ± 0.009	0.144 ± 0.013
LVd Mass (g)	0.643 ± 0.003	0.658 ± 0.006*
LVs Mass (g)	0.651 ± 0.004	0.668 ± 0.007*
HR	566.927 ± 22.789	554.321 ± 30.37

IVSd: Interventricular septum thickness at end-diastole, LVIDd: Left ventricular internal dimension at end-diastole; LVPWd: Left ventricular posterior wall thickness at end-diastole; IVSs: Interventricular septum thickness at end-systole; LVIDs: Left ventricular internal dimension at end-systole; LVPWs: Left ventricular posterior wall thickness at end-systole; SV: Stroke volume; LVd Mass: LV mass at end diastole; LVs Mass: LV mass at end systole. Data are shown as mean ± SEM, *$P < 0.05$, Student's t-test.

Table S5: Echocardiography data of H19 knockout mice after MI.

**Parameters**	**H19$^{+/+}$ (MID4)**	**H19$^{-/-}$ (MID4)**
No. of mice	10	10
IVSd (mm)	0.373 ± 0.002	0.340 ± 0.002
LVIDd (mm)	5.036 ± 0.008	4.64 ± 0.008**
LVPWd (mm)	0.364 ± 0.002	0.35 ± 0.003
IVSs (mm)	0.555 ± 0.002	0.57 ± 0.002
LVIDs (mm)	4.191 ± 0.008	3.78 ± 0.008**
LVPWs (mm)	0.536 ± 0.002	0.5 ± 0.003
SV(ml)	0.124 ± 0.005	0.109 ± 0.006
LVd Mass (g)	0.654 ± 0.003	0.645 ± 0.003
LVs Mass (g)	0.662 ± 0.002	0.651 ± 0.003*
HR	579.962 ± 16.674	564.669 ± 29.032

IVSd: Interventricular septum thickness at end-diastole, LVIDd: Left ventricular internal dimension at end-diastole; LVPWd: Left ventricular posterior wall thickness at end-diastole; IVSs: Interventricular septum thickness at end-systole; LVIDs: Left ventricular internal dimension at end-systole; LVPWs: Left ventricular posterior wall thickness at end-systole; SV: Stroke volume; LVd Mass: LV mass at end diastole; LVs Mass: LV mass at end systole. Data are shown as mean ± SEM, $*P < 0.05$, $**P < 0.01$, Student's t-test.
